# The Drivers of the Martian Bow Shock Location: A Statistical Analysis of Mars Atmosphere and Volatile EvolutioN and Mars Express Observations

**DOI:** 10.1029/2021JA030147

**Published:** 2022-05-25

**Authors:** P. Garnier, C. Jacquey, X. Gendre, V. Génot, C. Mazelle, X. Fang, J. R. Gruesbeck, B. Sánchez‐Cano, J. S. Halekas

**Affiliations:** ^1^ IRAP Université de Toulouse CNES CNRS UPS Toulouse France; ^2^ ISAE‐SUPAERO Université de Toulouse Toulouse France; ^3^ Laboratory for Atmospheric and Space Physics University of Colorado Boulder CO USA; ^4^ Department of Astronomy University of Maryland College Park MD USA; ^5^ NASA Goddard Space Flight Center Greenbelt MD USA; ^6^ School of Physics and Astronomy University of Leicester Leicester UK; ^7^ Department of Physics and Astronomy University of Iowa Iowa IA USA

**Keywords:** Mars, bow shock, solar wind, LASSO, Akaike information criterion

## Abstract

The Martian interaction with the solar wind leads to the formation of a bow shock upstream of the planet. The shock dynamics appear complex, due to the combined influence of external and internal drivers. The extreme ultraviolet fluxes and magnetosonic Mach number are known major drivers of the shock location, while the influence of other possible drivers is less constrained or unknown such as crustal magnetic fields, solar wind dynamic pressure, or the Interplanetary Magnetic Field (IMF) intensity, and orientation. In this study, we compare the influence of the main drivers of the Martian shock location, based on several methods and published datasets from Mars Express (MEX) and Mars Atmosphere Volatile EvolutioN (MAVEN) missions. We include here the influence of the crustal fields, extreme ultraviolet fluxes, solar wind dynamic pressure, as well as (for MAVEN, thanks to magnetic field measurements) magnetosonic Mach number and Interplanetary Magnetic Field parameters (intensity and orientation angles). The bias due to the cross‐correlations among the possible drivers is investigated with a partial correlations analysis. Several model selection methods (Akaike Information Criterion and Least Absolute Shrinkage Selection Operator regression) are also used to rank the relative importance of the physical parameters. We conclude that the major drivers of the shock location are extreme ultraviolet fluxes and magnetosonic Mach number, while crustal fields and solar wind dynamic pressure are secondary drivers at a similar level. The IMF orientation also plays a significant role, with larger distances for perpendicular shocks rather than parallel shocks.

## Key Points

- The relative importance of the drivers of the Martian shock location is studied according to the AIC and LASSO model selection methods
- The Mach number and EUV fluxes are the primary drivers of the shock location, crustal fields, and solar wind pressure are secondary drivers
- The angle between the shock normal and the Interplanetary Magnetic Field appears as a significant driver of the shock location

## 1 Introduction

Due to the absence of a global dynamo magnetic field, the dynamics of the Martian environment are complex with a variety of drivers shaping the induced magnetosphere. The Martian interaction with the solar wind leads to the presence of a bow shock upstream of the planet, as well as several other plasma boundaries, that is, the Induced Magnetospheric Boundary or the PhotoElectron Boundary. The boundaries are dynamic and depend on internal and external drivers (see e.g., Garnier et al., 2017; Matsunaga et al., 2017), and studying their dynamics is crucial to better understand the evolution of the Martian environment with time.

In the literature, several drivers were considered for the Martian bow shock (hereafter BS) location, based on the data obtained by the Mars Global Surveyor (MGS), Mars Express (MEX), and Mars Atmosphere and Volatile EvolutioN (MAVEN) missions. The first driver analyzed was the solar wind (SW) dynamic pressure (Crider, 2004; Vignes et al., 2002). Edberg et al. (2010) later showed that the magnetosonic Mach number (Mms) of the SW influences significantly the BS. Hall, Lester, Sánchez‐Cano, et al. (2016) (hereafter BH16) analyzed MEX data and showed the BS location is more sensitive to seasonal variations in the solar extreme ultraviolet (EUV) irradiance than to SW dynamic pressure variations, and Hall et al. (2019) also showed the influence of solar cycle EUV dynamics. Halekas et al. (2017) confirmed the major influence of the magnetosonic Mach number and EUV based on MAVEN data, as well as a significant influence of SW dynamic pressure and a weak longitudinal dependence due to crustal fields.

Beyond these main drivers (EUV, *Mms*, and SW dynamic pressure), the influence of other potential drivers is less understood. In particular, the influence of the crustal field is poorly constrained by previous studies. Most of the works suggested a possible influence of the crustal fields, based on hemispheric differences between the north and south locations of BS (e.g., Edberg et al., 2008; Mazelle et al.,2004) or on the dayside versus nightside location of the strongest crustal source region located in the southern hemisphere (Gruesbeck et al., 2018). Overall, the influence of the crustal fields on the BS varies in the literature from no or little influence (Edberg et al., 2009) to strong variabilities, up to above 1,000 km influence based on North/South asymmetries (Edberg et al., 2008; Gruesbeck et al., 2018) and is considered through either a local impact (Nemec et al., 2020) or a global influence (Fang et al., 2015, 2017).

Garnier et al. (2022) ‐ hereafter referred to G22 ‐ analyzed in detail the influence of the crustal fields on the Martian BS location by combining datasets from MAVEN and MEX. They showed the influence of the crustal field is significant (with several hundreds of km of induced variation) with a primary influence of the strongest crustal field source region in the southern hemisphere, seen first through a clear dependence on the angular distance to this region. The BS location also varies with the crustal field pressure integrated over large areas, or with the planetary longitude when focusing around southern latitudes instead of considering all latitudes as in previous studies. Moreover, they showed modulation of this influence that maximizes when the strongest crustal source region is located closer to noon, with no clear influence observed beyond the terminator. They also found a lower limit of the crustal field pressure to observe a related modification of the BS location and showed that rapid rotations of the IMF apparently hide the influence of the crustal fields. They revealed the presence of a bias in the MAVEN data, due to a cross correlation between crustal fields regions and the EUV fluxes, which underlines that using the North/South asymmetry of physical parameters as a proxy for the crustal field influence as performed by several works provides incorrect estimations of this influence. Moreover, they provided a simple composite parameter that is representative of the overall behavior of the BS location with respect to EUV and magnetosonic Mach number (the two major drivers) as well as crustal fields. Finally, they showed the existence of seasonal variability of the influence of the crustal fields on the BS, strongly correlated to the MARSIS Total Electronic Content variability, which reveals the existence of a large scale coupling between the BS, the crustal fields, and the ionosphere.

The influence of the IMF parameters ‐ intensity, and orientation ‐ was widely discussed in the literature on the Venusian and terrestrial BS to explain BS asymmetries observed by several missions. The Venusian BS showed indeed evidence in the Pioneer era for significant influence of the IMF parameters on BS asymmetries in the VSE frame (Venus‐Solar‐Electric field coordinates, with *x* axis pointing to the Sun and *z* axis along the upstream solar wind motional electric field). Pole versus equator, dawn/dusk, or north/south asymmetries were observed depending on the authors and often interpreted as the result of the strong mass loading of pickup ions, or of anisotropies of the magnetosonic wave velocity (Alexander et al., 1986; Jarvinen et al., 2013; Khurana & Kivelson, 1994; Russell et al., 1988). At Earth, a number of authors also linked dawn/dusk or North/South BS asymmetries observed to the IMF orientation (Chapman et al., 2004; Dmitriev et al., 2003; Peredo et al., 1995; Wang et al., 2016). At Mars, few studies investigated the influence of orientation. Vignes et al. (2002) suggested the presence of a North versus South asymmetry (in the MSE frame), consistent with the idea of asymmetric mass loading by picked‐up oxygen ions (e.g., Fang et al., 2008), but the authors mentioned the need for uncoupling from other major factors. Later, Edberg et al. (2009) also suggested an influence of the convection electric field on the Martian shock location, however, based on a proxy of the IMF direction based on MGS data since MEX could not provide such information. Wang, Xie, et al. (2020) recently proposed a 3D parametric model of the Martian BS based on an MHD model, suggesting that the IMF components have differential effects, but neither EUV nor crustal fields were included in this model.

**Figure 1:**
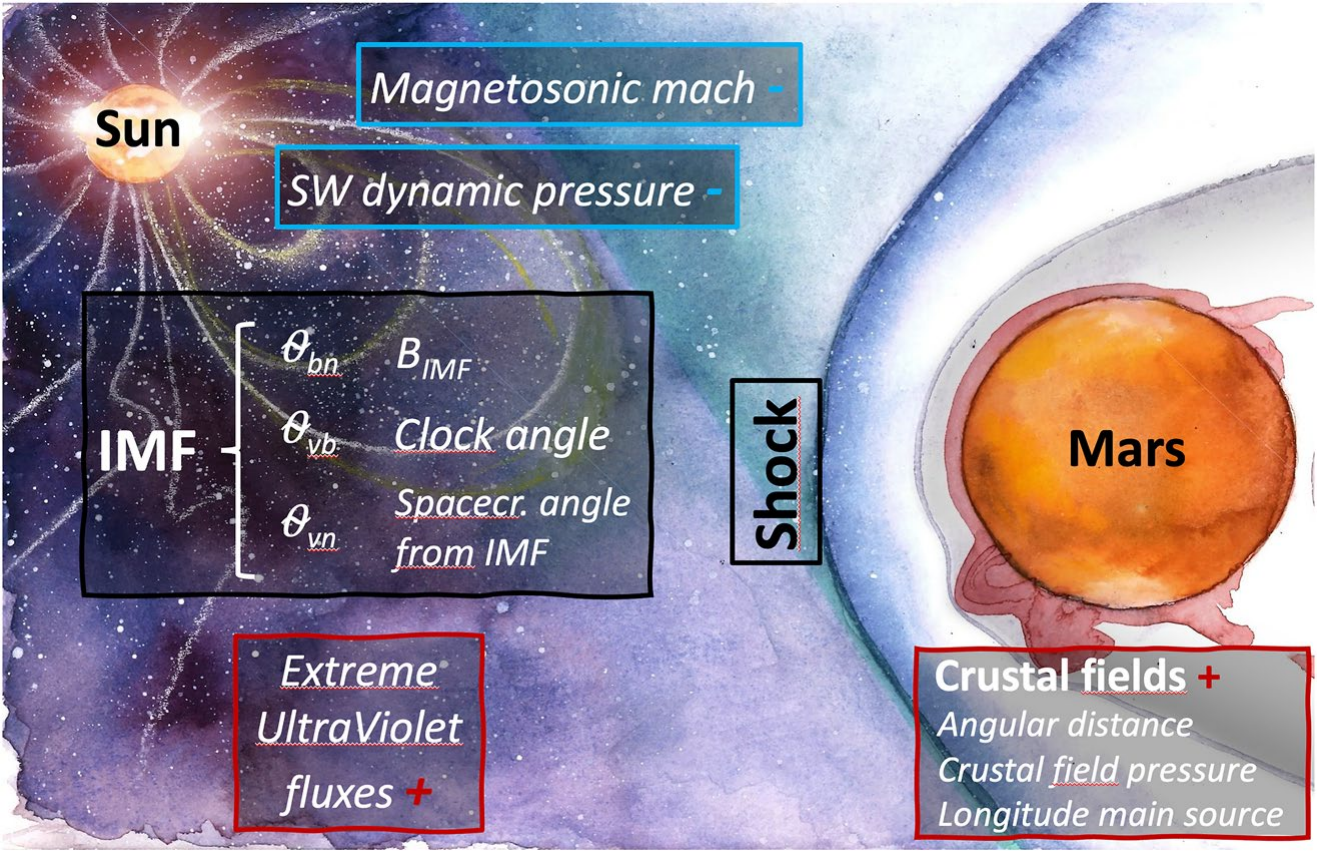
Schematics of the possible drivers of the Martian shock location considered in the paper (see text for explanations). *B*_*IMF*_ refers to the interplanetary magnetic field (IMF) intensity, *θ*_*bn*_ to the angle between the normal of the bow shock (BS) and the IMF vector, *θ*_*vn*_ between the normal of the BS and the SW velocity vector, *θ*_*bv*_ between the solar wind (SW) velocity vector and the IMF vector. The signs + (with red color) and − (blue color) refer to the known or expected impact of the driver, with increased crustal fields and Extreme UltraViolet fluxes pushing the BS further from the planet, while increased SW dynamic pressure and magnetosonic mach number push it closer to the planet. The background figure is a drawing by Anastasia Grigoryeva.

In this study, we investigate the possible drivers of the BS location based on both MAVEN and MEX BS crossings datasets. Instead of focusing on only few drivers (and independently from each other) as most authors, we compare and rank the relative importance of the drivers of the Martian BS based on several methods, including a partial correlation approach to take into account possible biases due to mutual correlation, and the Akaike Information Criterion (AIC) and Lasso regularization model selection methods to compare the relative importance of the drivers. This allows providing a comprehensive view of the Martian BS dynamics with respect to internal and external drivers. Figure 1 shows a sketch of the Martian interaction, with the possible drivers of the BS investigated in this work and detailed later: crustal fields parameters (angular distance from the strongest crustal field region center, crustal field pressure, longitude vs. Sun of the strongest crustal field region), solar Extreme UltraViolet fluxes, SW magnetosonic Mach number, SW dynamic pressure, IMF intensity and orientation (*θ*_*bn*_ angle between the normal of the BS and the IMF vector; *θ*_*vn*_ angle between the normal of the BS and the SW velocity vector; *θ*_*bv*_ angle between the SW velocity vector and the IMF vector, also called cone angle).

The paper is organized as follows: in Section 2 we first describe the MAVEN and MEX datasets used (2.1) as well as the methods considered in this study (2.2). Then, in Section 3, we first show the results of the direct analysis of the possible drivers of the Martian BS location (3.1) and then we show how cross‐correlations are considered to appropriately interpret the influence of minor drivers (3.2). A focus is then made on the influence of parallel versus perpendicular BS (3.4), before we compare the relative influence of the BS drivers with the Akaike Information Criterion or Lasso regularization techniques (3.5). We then end the paper with a discussion (Section 4) and a conclusion (Section 5).

## 2 Datasets and Methods

### 2.1 Description of the Datasets

We use in this study the same datasets as G22, which we refer to for a more detailed description of the lists of MAVEN and MEX crossings considered. Here we provide a brief description.

The MEX BS crossings dataset was derived by BH16 from the MEX ASPERA‐3 ELS data Barabash et al. (2006), including 11,820 crossings from January 2004 to May 2015. The MAVEN BS crossings dataset is a combination of the lists of crossings published by Fang et al. (2017) and Gruesbeck et al. (2018), derived from the analysis of the MAVEN magnetic field MAG data (Connerney et al., 2015), electron SWEA data (Mitchell et al., 2016), and ion SWIA data (Halekas et al., 2015). The MAVEN dataset comprises 3837 BS crossings from November 2014 to April 2017.

A large number of BS crossings for MEX and MAVEN ‐ due to their respective orbital periods of 6.7 and 4.5 hr ‐ allows for a large spatial and temporal coverage that enables statistical analyses. Significant differences between both datasets need to be considered. First, MEX covers a large period through a whole solar cycle, while the MAVEN dataset corresponds to a shorter period where the mean EUV level (given by the solar 10.7 cm radio flux) was larger by ∼14% compared with the MEX dataset. We thus focus on the dynamics rather than on absolute BS location due to the different solar conditions encountered. Second, the MEX spacecraft is not equipped with a magnetic field instrument, which prevents us from using MEX data to investigate the influence of IMF related parameters (such as IMF intensity and orientation, magnetosonic Mach number).

We derive and analyze the extrapolated terminator altitude already used by previous authors (Edberg et al., 2008; Fang et al., 2017; Hall, Lester, Sánchez‐Cano, et al., 2016). It allows to represent the variability of the BS location by removing the strong solar zenith angle influence, assuming an axisymmetric symmetry. The crossings location are transformed in the SW aberrated cylindrical MSO system (rotated by 4° for both MAVEN and MEX). The extrapolated terminator altitude, *R*_*TD*_, is calculated by:

(1) $$R_{TD}=\sqrt{L^{2}+\left(e^{2}-1\right)\cdot X_{0}^{2}+2\cdot e\cdot L\cdot X_{0}}-R_{M}$$

where *R*_*M*_ is the Martian radius (3390 km), *L* and *e* are the semi‐latus rectum and eccentricity, and the focus of the conic is located at (*X*_0_, 0, 0). We used for the MEX crossings the (*X*_0_, *e*) values by Hall, Lester, Sánchez‐Cano, et al. (2016), and for MAVEN the values by Fang et al. (2017). The choice of the conic parameters may introduce some uncertainties in the absolute values of *R*_*TD*_, but the variabilities basically remain unchanged in our experiments of using several conic fit parameters (e.g., Edberg et al., 2008; Fang et al.,2017; Hall, Lester, Sánchez‐Cano, et al., 2016) for both the MAVEN and MEX crossings. The impact of the conic parameters is discussed in Section 3.4.

Note the one dimensional approach allows the investigation of the presence of any large scale and permanent influence on the BS location, including asymmetries of the BS shape or a permanent influence of IMF intensity and orientation angles, as studied by numerous previous authors (e.g., Alexander et al., 1986; Chai et al., 2014, 2015; Edberg et al., 2009; Russell et al., 1988; Vignes et al., 2002; Wang, Lu, et al., 2020; Zhang et al., 1991); let us consider, for example, a strong local time asymmetry or further shocks at low cone angles: propagating the location of the respective crossings at dawn versus dusk or low versus large cone angles would lead to very different values of the distance to the conic focus, resulting in very different values of the recalculated semilatus rectum *L* (that depends on the distance from the focus), which would finally lead to very different *R*_*TD*_ values according to the above equation.

### 2.2 Description of the Methods

#### 2.2.1 Zero‐Order Correlations and Partial Correlations

Beyond the direct analysis of the extrapolated terminator altitude, we use in this paper correlation approaches defined below (for more details, see G22): zero‐order linear Pearson correlation coefficients, unpaired *t*‐tests and partial correlations.

First, simple linear Pearson correlation coefficients are used to investigate linear relationships between two variables. Hypothesis tests are used to calculate the significance of the correlation. A *t*‐test evaluates the test statistics associated with the correlation, and compares it with the statistics of the null hypothesis *H*_0_: if the test statistics *t* is larger than a threshold (i.e., the null hypothesis statistics $t_{H_{0}}$) defined for a given risk, or equivalently if the p‐value is smaller than the risk considered (5% by default), then the correlation is considered significant, otherwise the correlation factor is considered not significantly different from 0. We will in the rest of the paper mention that p‐values are negligible when they are smaller than 10^−5^ and tag them with ”(n)”. Unpaired *t*‐tests can also be used to determine if there is a significant difference between two groups of data. They assess whether the two groups show different mean values, with p‐values providing the significance of this difference. Throughout the paper, correlations factors, as well as significance tests statistics or p‐values by default, corresponding to direct linear correlations.

Second, we use a partial correlation approach in Section 3.2 to investigate possible biases due to cross correlations between parameters, and thus more appropriately quantify the influence of the drivers. The partial correlation approach (see Baba et al. [2004] or Appendix A of G22) allows to investigate of linear relations, calculating correlation coefficients ‐ and estimating their significance ‐ between for example, two variables *y* and *x*_0_, after controlling for the influence of other variables *x*_*i*_. G22 successfully used this technique to disentangle the influence of the crustal fields on the Martian BS location despite a significant cross correlation with EUV in the MAVEN data. It was also used by several authors for the solar wind Earth interaction (Simms et al., 2021), solar physics (Le & Zhang, 2017), or galaxies and compact objects (Ni et al., 2020; Yesuf & Ho, 2019). This method allows investigating cross correlations in a rigorous manner, without using sub‐selections of the dataset to control specific parameters, which reduces the number of samples in each dataset by a factor 2^*n*^ (n number of variables) which becomes critical in the case of the Martian BS that may be influenced by a number of parameters.

The correlations are considered linear (*y* = *a* + *∑*_*i*_ *x*_*i*_ ⋅ *b*_*i*_ with *a* constant and *b*_*i*_ slopes), but can alternatively correspond to power laws $\left(y=a\prod_{i}x_{i}^{b_{i}}\right)$ then linearized by a logarithm. Note that the linear assumption is weak and our method does not need true linear relationships to remain valid since at first order most of the regular relationships can be considered as linear (or power law like). The significance of the partial correlation factors is given by a *t*‐test to compare the statistics with the null assumption *H*_0_, and provides p‐values that need to be smaller than the risk chosen (5% by default) to consider the partial correlation as significant. The 5% risk level corresponds to a 2 standard deviations tolerance interval for a gaussian probability distribution.

#### 2.2.2 The Akaike Information Criterion and Lasso Regularization

Understanding the dynamics of the plasma boundaries implies understanding both the relationship with individual drivers (as can be performed through correlations, or partial correlations to minimize biases) and the relative influence of each of these drivers. Ranking the relative importance of the drivers can be achieved by model selection approaches such as the Akaike information criterion or the LASSO method.

The Akaike information criterion (AIC; see more details in Appendix A) is a model selection method based on information theory that allows to rank of the variables influencing a specific parameter (Akaike, 1974). Biesiada (2007) for example, used the AIC approach to rank competing dark energy models based on supernovae data, while Kubo (2008) compared several models for the probability density functions of solar flare interval, in order to develop probabilistic solar flare forecasting for space weather applications. In our case, the model considered is a linear regression (or a power law regression as described above) including a number of possible drivers of the BS (Section 3.5).

The Least Absolute Shrinkage Selection Operator (LASSO; see a detailed description in section Appendix B) is the second model selection approach that is used in Section 3.5 to compare the relative importance of the BS location. LASSO is one of the most commonly used supervised regularization method for regression (Tibshirani, 1996), where a penalty term allows the identification of the significance of predictors in a regression model. In a similar manner as in classical linear regression, it minimizes the residual sum of squares to determine the regression coefficients of each predictor, but here by penalizing the sum by the absolute values of the regression coefficients. LASSO is widely used in a variety of domains, such as space weather for solar flare detection/prediction (Benvenuto et al., 2018, 2020), identification of explanatory variables of supernovae magnitude (Uemura et al., 2015), or star formation model selection and stellar parameters estimation (Dey et al., 2019; Lu & Li, 2015).

We use in Section 3.5, the AIC and LASSO approach to investigate the relative importance of the Martian BS location drivers. These methods allow for selecting the best model with the smallest number of predictors by applying recent statistical techniques and avoiding overfitting compared with common multivariate regression models. Our aim is not to provide detailed functional forms of the Martian BS *R*_*TD*_ variability with respect to its drivers (which would need a theoretical description of these influences, and are not directly derived from either AIC or LASSO methods), but to provide a better understanding of the drivers of the BS location, and compare their relative importance.

## 3 Results

### 3.1 Direct Analysis of the Drivers Influence

In this section, we study the influence of the following possible drivers for the BS location, previously mentioned in Section 1 and derived as detailed below:

1. Solar Extreme UltraViolet fluxes (EUV) for MAVEN and MEX: the EUV values were determined for MAVEN from the FISM model (Chamberlin et al. [2007]; available for MAVEN but not MEX on the CDPP/AMDA database ‐ http://amda.cdpp.eu/) for 10–120 nm wavelengths, and for MEX from the solar 10.7 cm radio flux index extrapolated to Mars assuming a $1/r_{Mars\;Sun}^{2}$ law; using solar radio flux values for MAVEN leads to almost identical results since both parameters are strongly correlated (with a correlation factor of 0.98 (n)); however, we choose the FISM model for MAVEN since it is available and slightly more precise than the radio flux proxy, and the absolute values of EUV are of no interest in our study where we focus on methods which are unit independent
2. SW dynamic pressure for MAVEN and MEX: the SW dynamic pressure is derived from the SW parameters (plasma density, velocity) provided by respectively MEX/IMA and MAVEN/SWIA data; a restriction on the quality factor values for MEX/IMA data is applied, which reduces the number of MEX crossings available with this driver (this explains why the median *R*_*TD*_ value for SW dynamic pressure in Figure 3 discussed below is different from the others)
3. SW magnetosonic mach number (Mms) for MAVEN: the MAVEN *Mms* (no magnetic field measurements were available for MEX) is calculated by $Mms=v_{sw}/\sqrt{c_{s}^{2}+v_{A}^{2}}$ with *v*_*sw*_ SW speed, *c*_*s*_ sound speed, *v*_*A*_ Alfvén speed, with the electron temperature assumed equal to the proton temperature and a polytropic index *γ* = 5/3
4. crustal magnetic fields for MAVEN and MEX: several parameters for the crustal field were used and derived in particular from the Morschhauser et al. (2014) model at 400 *km* altitude, directly based on the results by G22:
  4.1. crustal field pressure (*B* ^2^/(2*μ*_0_)) at the sub‐spacecraft longitude/latitude of the crossing
  4.2. crustal field pressure averaged over an angular range of 75° around the local coordinates to account for the large spatial extent of crustal fields effects (the angular range value of 75° provides the strongest correlation between the crustal field pressure and the shock variability according to G22)
  4.3. averaged crustal field pressure convolved with a Gaussian‐like weighting function (with *σ* = 60°, based on G22), to increase the local influence of crustal fields in the averaging procedure
  4.4. angular distance to the strongest crustal source region located in the southern hemisphere (assumed centered at ∼−45° latitude and ∼180° longitude) to focus on the influence of the strongest crustal source region
  4.5. MSO longitude (*ϕ*_*MSO*_) of this strongest crustal source region to account for the larger impact of crustal fields when this crustal fields region is on the dayside
5. IMF intensity and orientation for MAVEN: the IMF vector is measured by the MAVEN MAG instrument, allowing to derive several parameters
  5.1. IMF intensity
  5.2. the clock angle of the IMF: $\tan^{-1}\left(B_{Z_{\mathrm{IMF}}}/B_{Y_{\mathrm{IMF}}}\right)$
  5.3. *θ*_*bn*_ angle between the normal of the BS (calculated from the axisymmetric shock shape based on the conic parameters by Fang et al. (2017) for MAVEN) and the IMF vector; this angle is used to determine whether BS is”parallel” (low *θ*_*bn*_ values) or perpendicular (large *θ*_*bn*_ values)
  5.4. *θ*_*vn*_ angle between the normal of the BS and the SW velocity vector
  5.5. *θ*_*bv*_ angle between the SW velocity vector and the IMF vector, also called cone angle
  5.6. the MSE pole versus equator asymmetry, determined by the absolute cosine of the angle between the IMF and the location of the crossing projected in the terminator plane (equivalent to the MSE frame representation); this angle was called ”clock angle” of the crossing by several authors for studies of Venus (Alexander et al.,1986; Russell et al., 1988) and can be used to show the influence of the mass loading of planetary ions picked up by the electric field, with possible enhanced BS location asymmetry in the direction perpendicular to the IMF

Note we use cosine or sine of the angles considered in this study to use comparable parameter ranges, but the use of direct angles leads to unchanged qualitative results.

**Figure 2:**
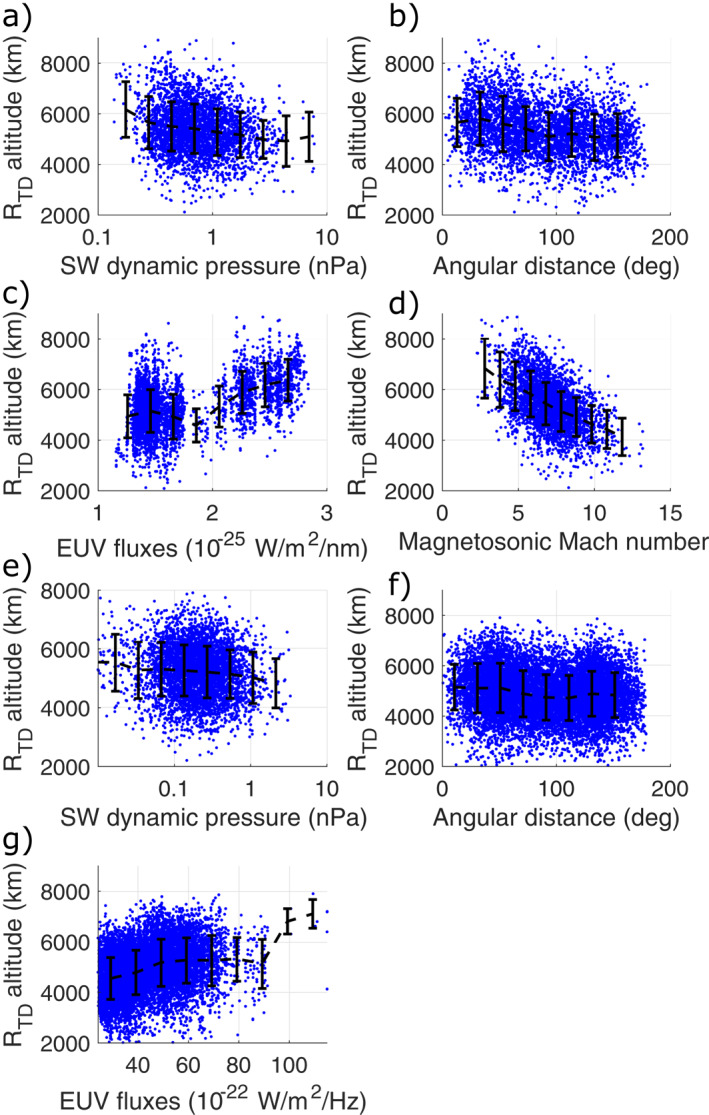
*R*_*TD*_ terminator altitude of the Mars Atmosphere Volatile EvolutioN (MAVEN) (panels a to d) and Mars Express (panels e to g) shock crossings as a function of: the solar wind dynamic pressure (panels a and e), angular distance of the spacecraft from the strongest crustal source region (panels b and f), Extreme UltraViolet fluxes (panels c and g), magnetosonic Mach number (for MAVEN only, panel d).

Figure 2 shows the MAVEN and MEX extrapolated terminator altitudes of the BS crossings as a function of the drivers considered significant in the literature: EUV, *Mms* (for MAVEN only), SW dynamic pressure, and the crustal fields through the angular distance from the strongest crustal source region introduced by G22. As shown by previous authors, the BS is located at higher altitudes in response to stronger EUV fluxes or lower *Mms* values, as well as under weaker SW dynamic pressures or spacecraft locations closer to the strongest crustal source region in the southern hemisphere. The MAVEN panels (upper panels) clearly show that larger *Mms* values lead to smaller *R*_*TD*_ values. Enhanced EUV leads to higher BS altitudes for both MAVEN and MEX datasets. The influence of SW dynamic pressure and angular distance to the strongest crustal source region is also visible in the observations by both missions: the mean profile (black dashed line) shows that despite very strong data scattering (due to the presence of other significant drivers at the same time and possibly spatial asymmetry of the boundary) the MAVEN and MEX BS *R*_*TD*_ values decrease with increasing SW dynamic pressure by hundreds of km; the panels b and f show the same results as G22, with decreasing *R*_*TD*_ values (by several hundreds of km) from the region around the strongest crustal fields ‐ with an angular extent of 40/50° corresponding to the size of the strongest crustal source region ‐ to regions away from the strongest crustal source region, with slight peaks around angular distances of 120–150° that may correspond to the influence of localized crustal sources indeed present at such angular distances. Note that the ranges of SW dynamic pressures are different between both missions: this may be due to either different time periods, or to the different types of instruments and methods used to extract the dynamic pressure; however, even if a real difference was existing, this would not impact our conclusions that are based on the variability of the drivers, with analysis for both missions that independent from each other.

**Figure 3:**
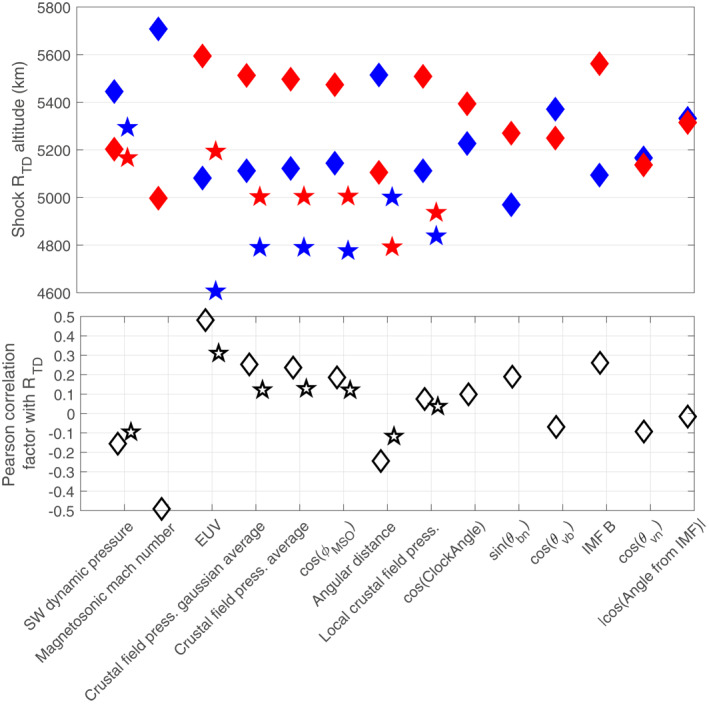
Comparison of the influence of a number of physical parameters on the shock *R*_*TD*_ terminator altitude as measured by Mars Atmosphere Volatile EvolutioN (diamonds; dataset published by Gruesbeck et al. [2018] and Fang et al. [2017]) and Mars Express (stars; dataset published by Hall, Lester, Nichols, et al. [2016]). See text for details.

In order to show the influence of the possible drivers of the BS altitude comprehensively and comparably, Figure 3 shows a simple and classic approach, based on direct correlation coefficients and *R*_*TD*_ values (see details below) for both MAVEN/MEX missions.

The figure provides two different ways of comparison between the various drivers. The lower panel provides the linear Pearson correlation coefficients between the terminator altitudes of the BS and the parameters. The upper panel uses the following approach: each set of parameters (EUV, Mms, etc.) was separated into low (=below the median value of the parameter) and high (=above the median value of the parameter) subsets; the median terminator altitudes of the ”low” (blue) and ”high” (red) subsets are then determined for each parameter to show how low/high regimes of each driver influences the terminator altitude of the BS.

The main differences between both missions are the smaller changes observed (in both altitude variations and correlations) for MEX compared to MAVEN, and lower terminator altitudes for MEX. Regarding the first difference, the much larger dataset for MEX (11,820 crossings compared to 1,760) induces a stronger mixing of different influences and a greater difficulty to distinguish one driver from another. Systematic differences may also exist between MAVEN and MEX due to different instruments and methods for determining the BS locations. The second difference most probably originates from the EUV levels encountered at the MAVEN and MEX crossings. The large MEX dataset allows for a wide range of EUV conditions, with a full solar cycle (including the lower and extended ever recorded solar minimum), while the MAVEN dataset corresponds to a period with an active Sun in 2014 and 2015 (where most of our MAVEN crossings occurred) below a declining activity in 2016–2017. The mean EUV level of the MAVEN crossings (given by the solar 10.7 *cm* radio flux: 49 ⋅ 10^−22^ *W*/*m* ^2^/*Hz*) was thus larger by ∼14% compared with the MEX dataset (43 ⋅ 10^−22^ *W*/*m* ^2^/*Hz*). Given the major influence of EUV on the BS location (see below), the larger EUV conditions associated with the MAVEN dataset certainly contribute to the slightly larger BS distances observed (by ∼5%) for MAVEN than for MEX. The compared magnetosonic Mach number conditions, which also have a major influence on the BS location, may also have induced this absolute difference in the BS altitude between both missions, however, these conditions are not known precisely for the pre‐MAVEN period.

Overall, based on the correlation coefficients or absolute variations related to individual parameters, the BS terminator altitude seems to increase with (in a descending order of importance and for both MAVEN/MEX) (a) and (b) increasing EUV fluxes or decreasing magnetosonic Mach number (MAVEN case, MEX probably as well given the results by Edberg et al., 2010), (c) either over stronger crustal field regions or near perpendicular BS (large sine of *θ*_*bn*_) or stronger IMF intensity, (d) decreasing SW dynamic pressure, (e) large cosine of clock and cone angles, and (f) other angular parameters of the IMF (*θ*_*vn*_ or the MSE pole vs. equator asymmetry). The apparent relative influence of the (non‐IMF related) drivers is consistent between the two spacecraft, with smaller variabilities and lower absolute altitudes for MEX than for MAVEN due to a larger amount of data and the different EUV conditions. A small difference can however be noticed in the relative importance of crustal fields versus SW dynamic pressure or EUV from MAVEN to MEX, with crustal fields appearing slightly more influent in the case of MAVEN: this is due to mutual correlation between parameters, that is, between EUV and crustal fields as discussed in details by G22, or between EUV and SW dynamic pressure as detailed in the next section that discusses the cross correlations between parameters. The apparent strong influence of the IMF intensity will also be discussed.

Note that using the averaged crustal field intensity instead of the averaged crustal field pressure, or an even smaller power law index of the pressure such as ${\left(B^{2}/\left(2\mu_{0}\right)\right)}^{1/6}$ as used by XF17, also leads to similar results with close correlation factor values or absolute variations that may vary by ±10% maximum with no change of the above ranking conclusions.

An illustration of the complex dynamics of the Martian BS that involves a combination of various physical drivers is the comparison between the extreme BS locations observed and the extreme solar events encountered by the MAVEN and MEX missions, which shows a variety of situations. The maximum *R*_*TD*_ value (7,913 km altitude) for the MEX dataset was reached on 22 October 2014 and was induced by a combination of large crustal fields, a relatively low SW dynamic pressure, and most importantly one of the largest EUV conditions encountered. These extreme EUV conditions were related to the event consisting of M and X type solar flares originating from the same active region 12,192 from 19th to 22 October 2014, surprisingly not accompanied with coronal mass ejections (Sun et al., 2015; Thalmann et al., 2015). Over the 3 days of the solar flares event, all BS crossings *R*_*TD*_ values were among the 7% largest of the MEX dataset. The smallest *R*_*TD*_ value (1614 km altitude) for the MEX dataset was reached on 25 June 2010, at a period with very low EUV (but not among the extreme low values), relatively strong SW dynamic pressure, and over the strongest crustal source region. No extreme solar CME‐type event happened closely before this crossing to our knowledge, except for the slow CME mentioned by Manchester et al. (2017) but which happened probably too early (on 16 June 2010) to be related to the extreme low Martian BS crossing location.

Regarding the MAVEN BS dataset, neither the extreme low (on 4 March 2016; *R*_*TD*_ = 2016 km altitude) nor the extreme high (on 31 January 2015; *R*_*TD*_ = 9243 km altitude) BS terminator distances correspond to extreme solar events as listed by Lee et al. (2017) who provided a comprehensive overview of the space weather events during the first 2 years of the MAVEN mission. These two extreme BS locations were due to a combination of both strong/low EUV and magnetosonic Mach number, but not among the most extreme values. Another typical example of the complex influence of solar events on the BS is the early March 2015 period where MAVEN encountered one of the stormiest space weather conditions, with a series of a solar flare and CME activity (including a strong ICME impact on 8 March 2015). Despite extreme external conditions (in EUV, SW dynamic pressure) leading to strong consequences (Jakosky et al., 2015) on the plasma escape rate, formation of the diffuse aurora, magnetosheath dynamics, or the BS shape according to simulations, the magnetosonic Mach number still kept moderate during the event (i.e., between 5.6 and 7.2 during the strong ICME) which prevented the BS from a strong compression. Moreover, EUV enhancements induced by solar flares counteract the influence of solar wind dynamic pressure pulses related to concomitant CMEs. The Martian BS location variability thus appears complex, with a variety of internal physical drivers that influence its location at the same time, in contrast with the Earth's BS driven primarily by the SW dynamic pressure and in a secondary way by magnetosonic Mach number (Cairns et al., 1995). This difference is due to the presence of an intrinsic magnetosphere at the Earth: the large scale dynamo magnetic field defines indeed the size of the obstacle at Earth, while the martian obstacle is defined essentially by the thin ionized atmosphere, with EUV and crustal fields thus playing roles that are minor at Earth, all the more the presence of an intrinsic magnetic field creates a magnetopause at Earth that is known to shape the bow shock (Wang et al. (2016) which is not the case at Mars.

### 3.2 Investigating Cross Correlations With the Partial Correlation Approach

The previous section suggested that a number of parameters simultaneously play a significant role in the BS location, although the simple approach used above considers the parameters independently from each other. Investigating the detailed influence of the parameters of influence, in particular the minor drivers, needs to consider the possible intercorrelations between them regardless of whether they are physically meaningful. G22 for example, highlighted how strong EUV fluxes were observed at the time of BS crossings observations where the spacecraft flew over the strongest crustal field regions of the planet, a coincidence due to observation conditions and to the spacecraft orbit precession during the mission.

**Figure 4:**
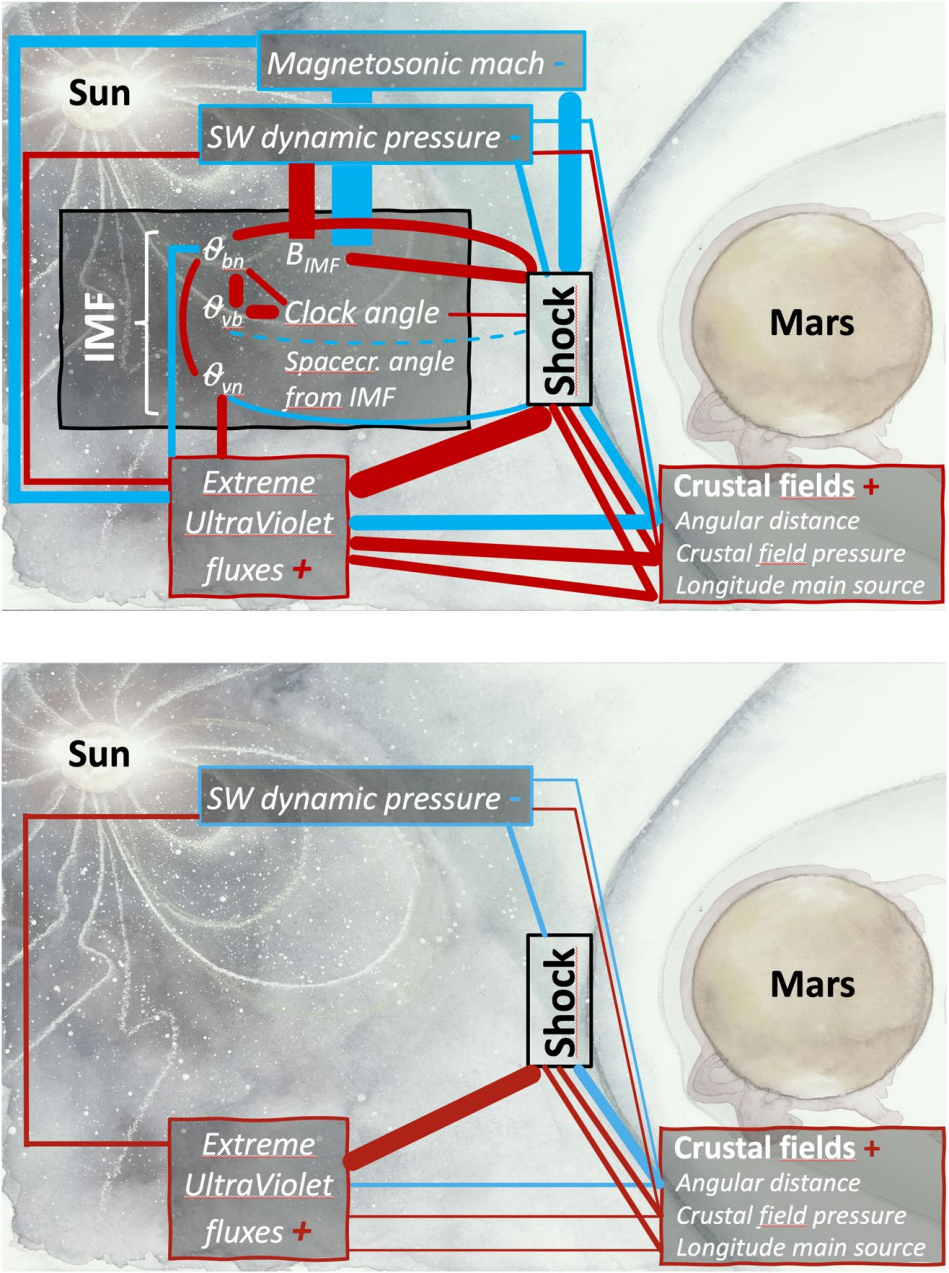
Schematic showing the complex inter‐correlations of a number of possible parameters of influence for the Mars Atmosphere Volatile EvolutioN (upper panel) and Mars Express (lower panel) shock terminator altitude. Blue and red lines correspond respectively to negative and positive Pearson linear correlation factors. The thickness of the lines is proportional to the Pearson correlation factor. Dashed lines represent non‐significant (*p*‐value above 5%) correlations. The background figure was adapted from a drawing by Anastasia Grigoryeva.

Figure 4 shows a diagram of how physical parameters that potentially influence the Martian BS location may be intercorrelated. The figure shows the same figure as Figure 1 where we superimposed the direct linear Pearson correlation factors between the BS terminator altitude and the possible drivers of the BS location, as well as between the possible drivers themselves. The thickness of the lines is proportional to the correlation factor, while the color gives the sign of the correlation.

The schematics show, beyond the statistically significant correlation (p‐values below 5%) between a number of drivers and the BS location (with in particular the major influence of EUV and Mms), cross correlations between most of the drivers themselves. One notes that the direct correlations between the major drivers and the BS *R*_*TD*_ are stronger than the cross correlations, which increases the confidence in their direct influence on the BS location. For example, the EUV correlation with *R*_*TD*_ is stronger than the correlation of EUV with other possible drivers. This is the case for EUV (with both MEX and MAVEN datasets), *Mms* (MAVEN dataset), and crustal fields (with the MEX dataset, see below for the MAVEN dataset).

The cross correlations among the drivers must be considered to avoid biased interpretations, particularly when they are comparable or even greater than the direct zero‐order correlation with *R*_*TD*_. In the case of the MAVEN dataset, the cross correlations between the crustal field parameters considered and the BS location is for example, smaller than the cross correlations between the crustal field parameters and the EUV. At least a part of the influence of the crustal fields in the MAVEN dataset shows up through the cross influence of EUV which is a major driver of the BS location. G22 investigated this issue and used the partial correlation approach to investigate whether the crustal fields had a significant influence not only in the MEX dataset (where no such bias exists) but also in the MAVEN dataset. They concluded that indeed a part of the apparent crustal fields' influence was due to the cross correlation with the EUV conditions, but controlling for the EUV influence in the analysis still leads to very significant partial correlations of the angular distance to the strongest crustal source region or of *ϕ*_*MSO*_ the MSO longitude of the strongest crustal source region center with the BS *R*_*TD*_ (with negligible p‐values of respectively 10^−11^ and 10^−7^). Controlling over the EUV then leads to similar correlations factors and similar relative influence of the EUV and crustal fields parameters between MEX and MAVEN based on the slopes of the multivariate regression performed with the partial correlation analysis.

Even when cross correlations are not strong enough to interfere with the influence of a driver, they can lead to an overestimation or an underestimation of the influence of the drivers. If one considers two variables *x*_1_ and *x*_2_ that influence the parameter *y* (in our case *R*_*TD*_) and that can be cross correlated to each other, three correlation factors can be considered: *r*_12_, *r*_1*y*_ and *r*_2*y*_. When the product of the signs of the three correlation factors is positive (e.g., *r*_12_ > 0, *r*_1*y*_ < 0, and *r*_2*y*_ < 0), this means the influence of variables *x*_1_ and *x*_2_ on *y* is to some extent overestimated when considering only the direct correlation factors *r*_1*y*_ and *r*_2*y*_, while their influence is underestimated when the product of the signs of the correlation factors is negative. Depending on the relative signs of the correlations, a part of the increase or decrease of *y* due to a correlation with one of the variables (e.g., *x*_1_) may be indeed partially due to or hidden by the increase of the second variable (e.g., *x*_2_) that is not only correlated with *y* but also with *x*_1_. This can be illustrated by the example of the influence of the SW dynamic pressure, as detailed below.

The SW dynamic pressure influence on the BS location is clearly visible when simple scatter plots with the BS location are performed, with in particular fewer high altitudes crossings at pressures above 1 *nPa* (in a similar manner to the MGS results by Crider et al. (2003)). However, the (linear or power law) negative correlation with *R*_*TD*_ is relatively small (−0.12/−0.10 (n) with a linear assumption for MAVEN/MEX) compared to the correlations of *R*_*TD*_ with EUV fluxes (strong positive correlation) or magnetosonic Mach number (strong negative correlation), and also smaller than correlations with crustal field pressure or angular distance. However, these direct correlations of the SW dynamic pressure previously mentioned in Section 3.1 may be slightly underestimated in both MAVEN and ‐to a lesser extent ‐ MEX datasets due to a small positive cross correlation between SW dynamic pressure and EUV fluxes (correlations of respectively 0.12 (n) and 0.09 (n) for MAVEN and MEX datasets): the solar wind dynamic pressure was for example, for MAVEN on average of ∼0.8 *nPa* during the low EUV periods, and of ∼1 *nPa* during the high EUV periods. Enhanced EUV fluxes push the BS to higher altitudes, so that enhanced EUV fluxes occurring at the same time as slightly enhanced solar wind dynamic pressure may partially hide the compression of the BS due to small solar wind dynamic pressure pulses. With a partial correlation analysis assuming a linear regression (or power law with similar results) and control over the EUV fluxes and ‐ for MAVEN only ‐ magnetosonic Mach number, the partial correlations between the solar wind dynamic pressure and the BS *R*_*TD*_ altitude become larger at −0.24(n) and −0.14(n), respectively, for MAVEN and MEX. After controlling for cross‐correlations with the major drivers, the solar wind dynamic pressure thus appears as a driver with a similar influence (regarding correlation factors) as the crustal field pressure or angular distance.

**Figure 5:**
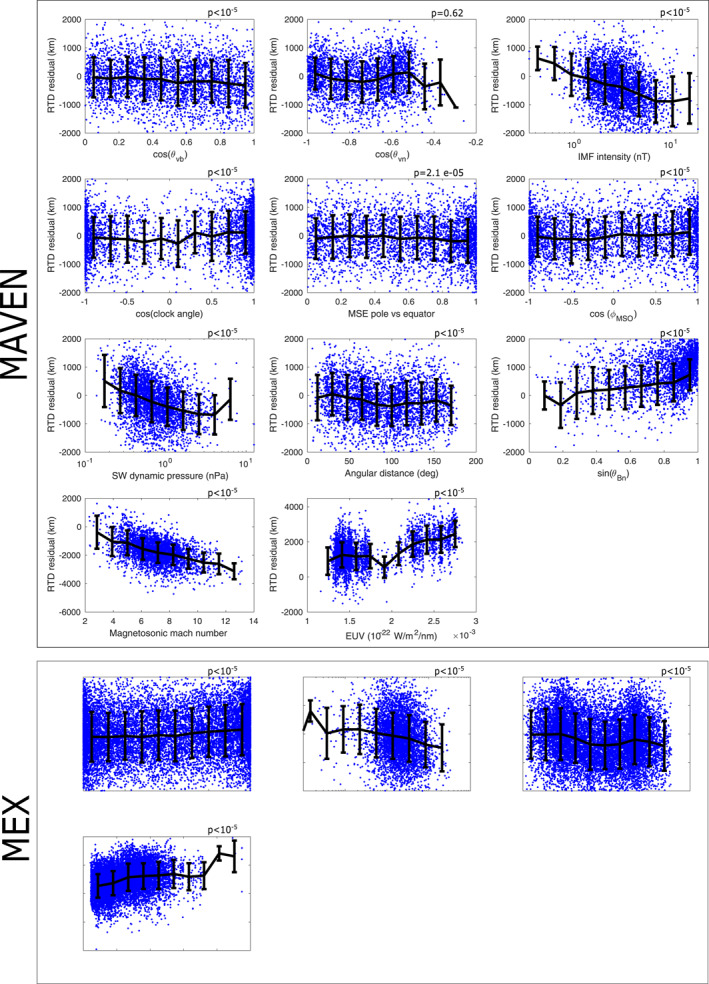
Residuals of the *R*_*TD*_ terminator altitude (in *km*) of the MAVEN (upper panels) and Mars Express (lower panels) shock crossings versus the possible drivers of the shock location, after removing the linear dependance versus the main drivers. The main drivers considered to calculate the residuals are the magnetosonic mach number (only available for MAVEN) and the Extreme Ultraviolet fluxes. Numbers above the panels correspond to p‐values associated with the linear partial correlations (where <10^−5^ refers to negligible p‐values).

We show in Figure 5 the residuals of the *R*_*TD*_ terminator altitude of the MAVEN and MEX BS crossings versus candidate drivers, after removing the linear dependence on the major drivers (EUV and *Mms* for MAVEN, EUV for MEX). The resulting p‐values of the partial linear correlation coefficients are also shown. After controlling for the major drivers of the BS location, most of the possible drivers still keep a significant correlation with the BS location. This includes the crustal fields (described by the independent variables that are angular distance and *ϕ*_*MSO*_ MSO longitude of the strongest crustal source region center), SW dynamic pressure, as well as the IMF intensity and several IMF orientation angles (*θ*_*bn*_, *θ*_*vb*_, clock angle, but not *θ*_*vn*_) and the MSE pole versus equator asymmetry, with correlations that are more or less obvious by eye (less for MSE pole vs. equator, *θ*_*vb*_ or clock angle). However, if these residuals confirm that a number of physical parameters can be possible secondary drivers of the Martian BS location, the schematics in Figure 4 underline the need to consider secondary drivers one by one, in particular the IMF and its orientation angles that are strongly cross correlated to each other and not only to the major drivers EUV and Mms. The IMF intensity appears also complex for example, a reversal of the influence on the BS location after controlling over *Mms* and EUV, which is discussed in the next section focusing on the influence of the IMF characteristics.

### 3.3 The Influence of the Interplanetary Magnetic Field

We focus on the MAVEN dataset in this section to analyze the influence of the IMF (intensity and orientation) on the Martian BS location. We discuss in more detail the impact of the IMF, whose characteristics include complex cross correlations beyond those with the major drivers. We also compare with results obtained in the literature at planets where the IMF influence was more widely investigated. The specific influence of the *θ*_*bn*_ angle is discussed in Section 3.4.

The IMF intensity is apparently significantly positively correlated with the *R*_*TD*_ altitudes (Pearson correlation factor of 0.26 (n)), in contradiction with the simple effect of an increased external magnetic pressure. A positive correlation could be due to the fact that an enhanced IMF induces a stronger draping around the planet, which could consequently push the BS further. However, Figure 4 shows that the IMF is even more strongly correlated with the SW dynamic pressure or the magnetosonic Mach number (Mms includes both parameters in its definition, with the IMF intensity through the Alfvén velocity). The control for both EUV and *Mms* (see Figure 5) thus changes strongly the apparent influence of the IMF intensity, with even a reversal of the correlation that becomes negative with the MAVEN BS *R*_*TD*_ (Pearson correlation factor of −0.21 (n)). Including also the SW dynamic pressure in the partial correlation in addition to EUV and *Mms* shows a much‐reduced correlation between the IMF and *R*_*TD*_ with a Pearson linear correlation coefficient of −0.07 (p‐value of 0.01%). The real influence (if any) of the total IMF intensity on the BS location thus is difficult to be distinguished due to complex mutual correlations with other significant drivers.

Wang, Xie, et al. (2020) recently proposed a 3D parametric model of the Martian BS based on a MHD model, including solar wind parameters (dynamic pressure, IMF intensity, and orientation, Mms) but not EUV or crustal fields. Beyond the strong influence of *Mms* (and of SW dynamic pressure according to the authors), they suggested that the IMF components have differential effects, with the BS expanding along the MSO *Y*/*Z* axis for respectively increasing *B*_*Z*_/*B*_*Y*_ IMF components and a stronger influence of *B*_*X*_ in the tail. However, the analysis of our dataset does not show such differential influence on the BS *R*_*TD*_ altitudes: the IMF components have little influence compared to the total IMF on *R*_*TD*_, with for example, a partial correlation significance of *B*_*Z*_ with *R*_*TD*_ below the null *H*_0_ assumption when focusing on BS crossings around the MSO *Y* axis sector. An influence of the IMF *B*_*X*_ component in the tail is however difficult to identify, due to the limited number of MAVEN crossings in the tail (only 24 crossings occurred at *X* < −1 *R*_*M*_).

Beyond the IMF intensity, the orientation of the IMF is often considered as a possible driver of planetary BS through various angular characteristics. First, the cosine of the angle *θ*_*vn*_ has an apparent small negative but still statistically significant direct correlation with the BS *R*_*TD*_ values, but controlling for the EUV fluxes (or for the other main drivers) with the partial correlation approach reduces the significance of the correlation well below the *H*_0_ null assumption (p‐value of 49%). Similarly, the significance of the already small correlation between the cosine of the cone angle *θ*_*bv*_ and the BS distance becomes non‐significant (p‐value of 31%) after controlling for the *θ*_*bn*_ influence. No significant cone angle dependence can thus be seen as was observed at Venus by Alexander et al. (1986), where the authors interpreted BS variations as possibly due to a more efficient mass loading through an enhanced conventional electric field $\vec{E}=-\vec{V}X\vec{B}$ cross product for incident flows perpendicular to the IMF. At Earth, Wang, Lu, et al. (2020) showed that the flaring of the Earth's BS increases at low cone angles, leading to further shocks in the tail and closer shocks near the nose, which is not observed with our martian dataset based on partial correlations and direct plotting of the location of the crossing.

The clock angle of the IMF appeared as a significant driver in the previous analysis based on direct correlations, with BS located farther in response to low clock angles, that is, for an IMF along the MSO *Y* axis, thus corresponding to an electric field along the *Z* axis. However, cautions must be taken regarding this possible influence for two main reasons: (a) the clock angle distributions are highly inhomogeneous with ∼70% of the crossings with an IMF lying within 30° around the MSO *Y* axis and (b) cross‐correlations modify the apparent influence of the clock angle of the IMF. The residuals of the *R*_*TD*_ dependance after controlling for both EUV fluxes and *Mms* already reduce the clock angle influence (Figure 4), but the cross correlation with the *θ*_*bn*_ angle (Figure 5) is even larger: a partial correlation analysis shows that controlling simply for the sine of *θ*_*bn*_ reduces significantly the remaining correlation between the clock angle (its cosine) and the BS location close to the significance limit (p‐value of 5.3%). An influence of the clock angle thus appears still possible but small, which agrees with the findings by XF17.

In the literature, a number of studies make use of the ”clock angle” of BS crossings to reveal IMF related asymmetries. This ”clock angle” of a crossing actually corresponds to the angle between the IMF and the location of the crossing projected in the MSO *Y* − *Z* plane, equivalent to the MSE frame representation. At Venus, Alexander et al. (1986) and Russell et al. (1988) showed based on Pioneer Venus Orbiter data that the terminator distance of the Venusian BS was maximum along the E‐field leading to apparent ”pole/equator” asymmetry in the MSE frame, attributed to either a consequence of mass loading of planetary ions (picked up by the electric field) or to the fast mode magnetosonic wave speed dependence on the IMF orientation. Later, Chai et al. (2014, 2015) used the ”clock angle” variability based on Venus Express measurements to reveal the presence of north versus south, pole versus equator, and dawn versus dusk asymmetries of the Venusian BS. Chai et al. (2014) first suggested that the underlying reason for the pole/equator and dawn/dusk asymmetries was the influence of the tangential (to the BS surface) component of the IMF. In their following work, Chai et al. (2015) showed a decrease of the pole versus equator asymmetry at low SZA, which led the authors to conclude this asymmetry was rather caused by the anisotropic magnetosonic wave speed whose influence is expected to increase with travel time (which itself increases with SZA). Earth models by Wang et al. (2016) showed a larger shock tail cross section in the direction perpendicular to the IMF, most probably due to the influence of the *θ*_*bn*_ angle on the magnetosonic wave speed. At Mars, Zhang et al. (1991) used Phobos magnetometer data from only 27 BS crossings and concluded about pole versus equator asymmetries were similar to the Venus case. Vignes et al. (2002) used the ”clock angle” variability of the Martian BS extrapolated terminator distance from MGS data to suggest the presence of a North versus South asymmetry (in the MSE frame), with larger *R*_*TD*_ values along the upward electric field, consistent with the idea of asymmetric mass loading of the magnetosheath by the picked‐up oxygen ions. This asymmetry appeared stronger at large cone angles, in agreement with an enhanced influence of the $\vec{E}=-\vec{V}X\vec{B}$ cross product. The authors however mentioned the need for uncoupling from other factors in the future and also advised to check whether this asymmetry was enhanced during solar maximum when EUV induced ionization is increased. Later, Edberg et al. (2009) also suggested the presence of larger martian shock *R*_*TD*_ values along the convection electric field direction than on the opposite side, however based on a proxy of the IMF direction based on MGS data since MEX could not provide such information.

**Figure 6:**
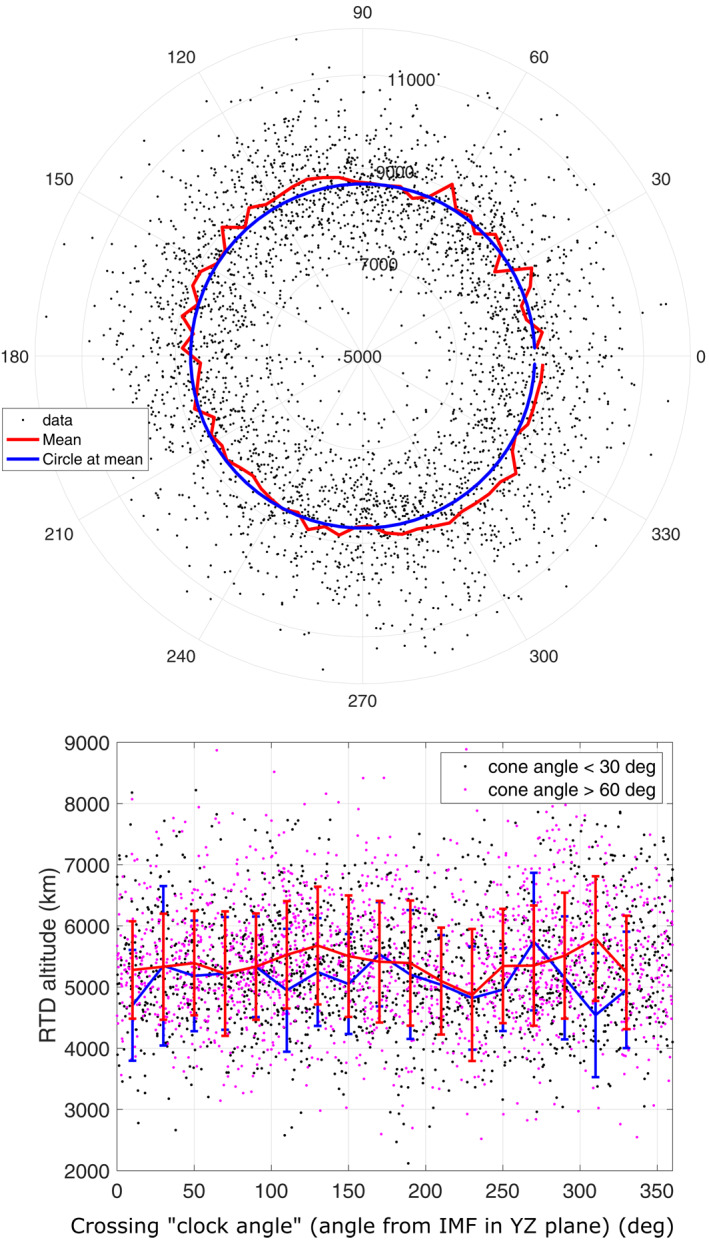
*R*_*TD*_ terminator altitude (in *km*) of the MAVEN shock crossings in the Mars Sun Electric field coordinate system, as a function of the angle between the projection in the terminator plane of the crossing and of the IMF vector (90° points toward the convection electric field). Individual crossings are given by black dots. (Upper panel) The averaged binned profile (red line) is compared with a constant profile at the overall mean value (blue circle). The radial axis starts from 5000 *km* altitude to focus on the variability around the mean. (Lower panel) Averaged mean profiles are superimposed for only large (>60°, red line) or low (<30°) cone angle values of the Interplanetary Magnetic Field. Both panels correspond to direct analysis as performed by previous authors, but can be biased due to cross correlations.

Figure 6 provides the MAVEN BS *R*_*TD*_ altitudes in the MSE frame (upper panel), as a function of the angle from the IMF vector in a radial way as performed by Russell et al. (1988), or in a linear way for low versus large cone angles (lower panel) as performed in numerous previous studies (Chai et al.,2014, 2015; Vignes et al., 2002; Wang, Lu, et al., 2020; Zhang et al., 1991). The upper panel does not show strong equator versus pole, or north versus south asymmetries. However, it suggests a small asymmetry with enhanced *R*_*TD*_ values along the axis defined by (120°, 300°) (thus similar to a polar vs. equator asymmetry but shifted by ∼30–45°), an asymmetry also suggested when focusing around close to terminator BS crossings only. The lower panel also suggests this asymmetry appears stronger at large cone angles, in agreement with an increased $\vec{V}X\vec{B}$ influence. Unpaired Student's *t*‐tests were performed and infirm the presence of a North versus South asymmetry suggested by Edberg et al. (2009) or Vignes et al. (2002) (even at high cone angles following the suggestions by Vignes et al. (2002)) or of a pole versus equator asymmetry (with large p‐values above 50%), but suggest the shifted pole versus equator asymmetry is a priori statistically significant (with p‐values of 3.3 ⋅ 10^−4^/5.6 ⋅ 10^−5^ for cone angles below/larger than 60°) before considering possible cross correlations. The partial correlation between the absolute cosine of the angle from the IMF and the BS location after controlling over EUV and *Mms* keeps significant (p‐value of 2.0 ⋅ 10^−5^, see Figure 5). However, a radial representation of the residuals shows little asymmetry. Moreover, the unpaired student *t*‐test mentioned above confirming an a priori shifted pole versus equator asymmetry becomes poorly significant (the p‐value rises up to 2%) as soon as we remove the EUV and Mms influence by considering residuals. A close to equator versus pole asymmetry is thus suggested by the MAVEN direct data analysis. Chai et al. (2014) mentioned that the Venusian BS equator versus pole asymmetry was stronger at the terminator or beyond for large IMF *B*_*Y*_ intensities, which is not observed with our dataset, but our tail coverage is poor. Overall, it is very difficult to conclude on the influence of a specific minor driver such as the ”clock angle” of the crossings based solely on a direct analysis such as provided in Figure 6, due to the combined influence of other important parameters (EUV, Mms…). Specific methods shall be used, such as partial correlations, AIC or LASSO (see Section 3.5), that are able to take into account the cross correlations between a number of possible drivers: their influence can indeed eventually hide or lead to an underestimation/overestimation of the influence of minor drivers when simple direct analysis is performed such as in Figure 6.

### 3.4 Quasi‐Perpendicular Versus Quasi‐Parallel Shocks

**Figure 7:**
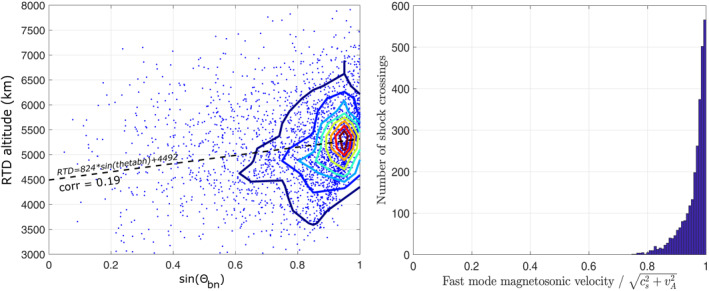
(left) *R*_*TD*_ terminator altitude (in *km*) of the MAVEN shock crossings versus the sine of *θ*_*bn*_ the angle between the IMF direction and the shock normal; contour (thick red) lines of the occurrence frequency and a linear regression (dashed line) are added, as well as the Pearson correlation coefficient between both parameters. (right) Histogram of the number of crossings as a function of the ratio between the fast mode magnetosonic wave velocity and.$\sqrt{c_{s}^{2}+v_{A}^{2}}$.

Another parameter suggested as significant in Section 3.1 is *θ*_*bn*_ the angle between the BS normal and the IMF direction, available for the MAVEN dataset. This angle is a distinguishing indicator between quasi‐parallel (low *θ*_*bn*_ values) and quasi‐perpendicular (large *θ*_*bn*_ values) BS. Our correlation analysis revealed a rather strong correlation (0.19 (n)) between the MAVEN BS *R*_*TD*_ altitude and the sine of *θ*_*bn*_ (or 0.22 (n) with *θ*_*bn*_ itself). Figure 7 shows the MAVEN BS altitude dataset organized as a function of the sine of *θ*_*bn*_ (*θ*_*bn*_ is considered positive). This profile suggests indeed that perpendicular BS happens on average at higher altitudes than parallel BS, which is also observed in the residuals of Figure 7 after removing the linear dependence with respect to EUV and Mms. We performed a partial correlation analysis that confirm the statistical significance of the *θ*_*bn*_ influence, with a large $t/t_{H_{0}}$ ratio above 8 (leading to negligible p‐value) when controlling over the magnetosonic Mach number, EUV and any of the other variables considered in this study (solar wind dynamic pressure or other IMF angle, angular distance…). Below, we investigate the physical reasons that could induce an influence of the *θ*_*bn*_ angle with perpendicular BS at higher terminator altitudes than parallel BS.

Several authors mentioned a similar influence at planetary BS, with further perpendicular BS than parallel BS, usually for the reason detailed below. The (Martian) BS is indeed a fast mode type of BS (Mazelle et al., 2004), with a fast mode magnetosonic wave velocity *ν*_*ms*_ defined by:

(2) $$\nu_{ms}=\sqrt{\frac{1}{2}\left[\left(c_{s}^{2}+V_{A}^{2}\right)+\sqrt{{\left(c_{s}^{2}+V_{A}^{2}\right)}^{2}-4c_{s}^{2}V_{A}^{2}\cos^{2}\theta_{Bn}}\right]}$$

with *c*_*s*_ sound speed and *v*_*A*_ Alfvén speed. Based on this equation, it appears that perpendicular BS (*θ*_*bn*_ close to 90°) leads to larger velocity values of the magnetosonic wave (the wave velocity being thus anisotropic), which could induce a further location of the BS.

In the literature, Alexander et al. (1986) or Russell et al. (1988) suggested at Venus that the asymmetries observed could be a consequence of an anisotropic magnetosonic wave velocity, instead of an increased mass loading effect. Khurana and Kivelson (1994) modeled this anisotropic velocity at Venus to account for an elliptic cross section of the BS in the tail. At Earth, Peredo et al. (1995) confirmed, based on a more than 1,000 BS crossings dataset issued from 17 spacecraft, previous analysis suggesting a north‐south versus east‐west asymmetry in the Mach cone. They suggested that the differential Mach cone extension was a consequence of the anisotropy of the magnetosonic wave velocity depending on the IMF orientation (through *θ*_*bn*_), with an influence mostly expected in the tail and for low Mach values (where the anisotropy has more time to develop). Dmitriev et al. (2003) then compared several BS models with Geotail and Wind crossings datasets (with >4000 crossings) and mentioned the important role of the dawn‐dusk asymmetry of the BS tail region that was mostly controlled by a faster magnetosonic wave due to a perpendicular configuration. Chapman et al. (2004) confirmed with MHD modeling the significant influence of the *θ*_*bn*_ angle due to enhanced wave velocity on the Earth's BS asymmetries, in particular at low Mach numbers. Later, Chai et al. (2015) analyzed the pole versus equator asymmetry observed at Venus and concluded on the influence of an anisotropic wave speed (due to *θ*_*bn*_) in particular since this asymmetry is reduced at low SZAs where the wave anisotropy is expected to lead to less influence than at higher SZAs where the BS is further and the travel time of the wave is larger. Wang et al. (2016) also investigated with MHD modeling the long debated influence of the IMF orientation on the Earth's BS. They showed that the enhanced magnetosonic wave velocity plays an important role in the tail asymmetries observed at low Mach numbers, with a maximum cross section in the direction perpendicular to the IMF. The Earth's BS thus appears controlled mostly by the solar wind dynamic pressure and the Mach number on the dayside, but the IMF orientation plays a major role in the tail with Mach number.

At Mars, a few early works also mentioned the influence of *θ*_*bn*_. Zhang et al. (1991) first used Phobos magnetometer data from 27 BS crossings to investigate the IMF control of the BS. They concluded that the Martian BS exhibited asymmetries similar to the Venus case in a magnetic frame (MSE equivalent), that is, North versus South, pole versus equator, and perpendicular versus parallel with respect to the IMF, the latter being presumably the consequence of an anisotropic wave velocity. Later, Vignes et al. (2002) mentioned a possible difference between perpendicular and parallel BS crossings (with perpendicular BS at *R*_*TD*_ values ∼3% above) but could not explain it. Overall, a number of Earth and Venus studies, as well as few Mars studies, thus mentioned a possible influence of the anisotropic wave velocity on the BS, essentially based on geometrical arguments rather than based on quantitative arguments.

However, in our case calculating the wave speed with Equation 2 reveals that it barely depends on the orientation of the IMF with respect to the BS normal. First, the wave speed appears essentially given by the combination of the sound and Alfvén speeds $\sqrt{c_{s}^{2}+V_{A}^{2}}$, with 90% of the MAVEN BS crossings showing a ratio $\frac{a_{\mathrm{fast}}}{\sqrt{c_{s}^{2}+V_{A}^{2}}}$ above 0.9. Second, one would expect, if an anisotropic wave speed was the reason for the *θ*_*bn*_ influence, that the BS *R*_*TD*_ altitude would be more strongly correlated with *ν*_*ms*_ rather than with *θ*_*bn*_ or its sine. The direct correlations of *ν*_*ms*_ and the sine of *θ*_*bn*_ with *R*_*TD*_ are actually similar (respectively 0.20 (n) and 0.19 (n)), but much of the influence of the wave speed on the BS location is due to the *Mms* influence through the Alfvén velocity that is closely related to the wave speed *ν*_*ms*_ (with a very strong correlation of −0.71 (n)): the partial correlation of *ν*_*ms*_ with *R*_*TD*_ becomes small and even slightly negative (−0.06) after controlling over the influence (assumed linear at first order) of EUV and (mostly) Mms. Similarly, the partial correlation between the sine of *θ*_*bn*_ and *R*_*TD*_ keeps strong and almost unchanged (correlation factor 0.16 (n)) when controlling for the fast magnetosonic wave speed value, thus suggesting an independent influence of the *θ*_*bn*_. The apparent influence of the wave speed is thus due to the magnetosonic Mach number influence rather than the IMF orientation. Moreover, the analysis of the influence of *θ*_*bn*_ shows no specifically stronger influence at low SZAs or low Mach number regimes that would be expected with an anisotropic wave speed influence (Chai et al., 2015; Peredo et al., 1995). One can note Chai et al. (2014) suggested that the influence of *θ*_*bn*_ on the differential perpendicular versus parallel BS at Venus was due to the influence of the tangential component of the IMF rather than the anisotropic wave velocity (the latter explanation being preferred in their following paper in Chai et al. [2015]). However, if the direct correlation of this tangential component with the BS location also appears strong in our dataset (correlation factor of 0.30 (n)), it is due to the cross‐correlation with *Mms* that is a major driver of the BS location: the partial correlation of the IMF tangential component with *R*_*TD*_ becomes very small (−0.03, with a p‐value of 2.3% only) after controlling over *Mms* and EUV, while the *θ*_*bn*_ influence does not reduce after controlling for a linear influence of *Mms* or other variables.

Apart from the anisotropy of the wave speed explanation, several authors linked the perpendicular versus parallel BS differences to dawn versus dusk asymmetries observed at planetary BS, in particular since the Parker spiral average configuration implies (depending on the IMF *B*_*X*_ component) that parallel/perpendicular BS correspond to respectively dawn/dusk sectors (see expectations at Earth by Walters [1964] and Dmitriev et al. [2003], or Vignes et al. [2002] results at Mars, and Chai et al. [2014] at Venus). Gruesbeck et al. (2018) mentioned possible dawn versus dusk asymmetry of the Martian BS based on MAVEN data, but could not explain its absence at low SZAs. Our analysis of the local time variation of the MAVEN BS *R*_*TD*_ values reveals a complex situation: dusk BS occurs indeed at higher altitudes at intermediate SZAs above 45°, but the situation reverses when reaching the terminator region, with overall a noisy profile that makes it difficult to conclude on a possible global dusk dawn asymmetry of the BS that could be related to the *θ*_*bn*_ influence. *t*‐tests and partial correlation analysis confirmed the absence of significant influence of local time at a global scale (with associated risks of non‐partial correlation above 5%). Moreover, the MEX dataset shows a reversed situation, with dawn BS occurring at further altitudes for intermediate SZAs until the terminator where the situation is reversed with dusk shocks at further altitudes than dawn shocks.

The distribution of the *θ*_*bn*_ angle values encountered in the MAVEN dataset is not Gaussian, with significantly more perpendicular BS than parallel ones: 80% of the crossings occurred at *θ*_*bn*_ values above 60°. It should be pointed out that this inhomogeneous sampling is not the result of a bias of detection of parallel BS being more difficult to identify from the data visual analysis: this *θ*_*bn*_ distribution is very similar to the distribution of the *θ*_*bn*_ values expected from the crossings by the MAVEN spacecraft with the modeled conic shape published by Edberg et al. (2008) ‐ and included in the 3DView webtool http://3dview.irap.omp.eu/‐ during the same period as our dataset. Note that updated crossing lists would not change the situation with more perpendicular than parallel BS encountered due to the spacecraft trajectory until 2021.

Parallel BS are usually more difficult to identify than perpendicular BS where the transition is more abrupt. In the case of perpendicular BS, one usually has a ’clean’ solar wind upstream with an absence of particles reflected. On the contrary, the transition at almost parallel BS is disturbed and wide, with a foreshock consisting of ions going up the solar wind (Meziane et al., 2017) and with different transients whose signatures may resemble those of a BS, including hot flow anomalies found at Mars (Collinson et al., 2015). The extension of the foreshock and the presence of transients could thus have resulted in an overestimation of the BS distance. However, our analysis shows that, on the contrary, perpendicular BS are located at higher altitudes than parallel ones.

A last possible uncertainty on the influence of the *θ*_*bn*_ angle on the BS location could be from the conic section assumption that is made in the derivation of the terminator distance *R*_*TD*_ in this study. In principle, if the eccentricity chosen was not appropriate with our dataset, extrapolating from the BS crossing location to the terminator could lead to an overestimation or underestimation of the *R*_*TD*_ value. However, this method is rather robust with respect to our conclusions. Let us choose only a subset of our dataset, that is, the list published by XF17 instead of a combination with the list by Gruesbeck et al. (2018), to keep consistent with the choice made of the conic parameters given by XF17. In this case the correlation of the sine of *θ*_*bn*_ with *R*_*TD*_ is unchanged (the Pearson correlation coefficient is changed by only 0.01 with negligible p‐value). If we keep the same dataset but use other conic parameters by Edberg et al. (2008) ‐ that is, (*X*_0_, *L*, *e*) = (0.55, 2.10, 1.05) instead of (0.42, 2.30, 0.87) for XF17 ‐ again the correlation factor is changed by only 0.01 with negligible p‐value. The choice of the conic parameters thus does not affect our observation of the *θ*_*bn*_ influence.

Finally, alternative explanations were provided by several authors to explain the presence of BS expansions. Thomas and Winske (1990) developed two‐dimensional hybrid simulations of planetary BS to study the foreshock ion population. They mentioned a similar asymmetry with the BS being closer on the quasi‐parallel side than on the quasi‐perpendicular side and considered this was a kinetic behavior since the asymmetry decreased during the simulations while the BS radius increased compared to the gyroradius. At Earth, magnetopause and BS expansions were also linked to radial (low cone angle) IMF configurations by several authors (e.g., Suvorova et al. [2010] or Wang, Xie, et al. [2020]), possibly due to the dependence of the magnetosonic wave velocity on the *θ*_*bn*_ angle. This is however not observed in our Martian dataset where the cone angle has no significant influence, with even slightly smaller BS *R*_*TD*_ values for radial IMF orientations.

The analysis of the MAVEN dataset thus shows that quasi‐perpendicular BS occurs at higher altitudes than quasi‐parallel ones, independently from the influence of other possible drivers. However, the exact origin of this influence is unclear. The common explanation of the fast magnetosonic wave speed dependence on the *θ*_*bn*_ angle of the IMF, suggested by a number of authors in the Earth and Venusian BS literature based essentially on geometric arguments, does not appear convincing in our quantitative analysis of the MAVEN data.

### 3.5 The Relative Importance of the Drivers

Understanding the dynamics of the plasma boundaries includes understanding both the relationship with individual drivers (as can be performed through correlations, or partial correlations to investigate biases) and the relative importance of each driver. The results discussed in Sections 3.1, 3.2, 3.3, suggested a ranking of the parameters of influence for the Martian BS location, but the methods used cannot appropriately quantify them. We use in this section two methods (described in Section 2.2), called Akaike information criterion and Lasso which are model selection methods that enable ranking.

#### 3.5.1 The Akaike Information Criterion

The Akaike information criterion (AIC; see Appendix A) is a method based on information theory that is dedicated to performing model selection (Akaike, 1974). AIC estimates, among several models fitting an observed dataset, the amount of information lost by each model to reproduce the data regularized by the dimension of the model. The model with a minimum AIC value is considered the best candidate (with no judgment on its absolute quality), and only the relative AIC difference value among the two models is meaningful to compare their relative likelihood.

We performed AIC calculations with a dedicated R code, assuming either a linear dependence of the Martian BS terminator distance *R*_*TD*_ with respect to several parameters (*RTD* = *a* + *∑*_*i*_ *X*_*i*_ ⋅ *b*_*i*_), or a power law dependence $\left(RTD=a\prod_{i}X_{i}^{b_{i}}\right)$. Table 1 shows the result of an AIC approach, where the AIC is calculated after each parameter of influence is removed one after the other in decreasing importance order (backward regression). AIC thus compares several models, each model including all drivers but one. For example, if EUV is removed from the MAVEN model including initially all drivers, the AIC value given is 37,049, which is larger than if IMF intensity is instead removed from the initial model (AIC value of 36,408): removing EUV leads to a model with more information loss compared to measurements, it is thus ranked as a more important driver than IMF intensity.

**Table 1:** Akaike Information Criterion Results for MAVEN and MEX Linear Regression Models of the Martian BS Terminator Distance

Variable removed from model	MAVEN AIC	MEX AIC
No variable removed	36,405	60,072
IMF intensity	36,408 (9)	
cos(*clockangle*)	36,410 (8)	
MSE pole versus equator	36,411 (7)	
cos(*ϕ*_*MSO*_)	36,419 (6)	60,105 (4)
SW dynamic pressure	36,461 (4)	60,171 (3)
Angular distance	36,423 (5)	60,174 (2)
Magnetosonic mach	36,626 (3)	
sin(*θ*_*bn*_)	36,661 (2)	
EUV	37,049 (1)	60,620 (1)

*Note.* The ranking number of each driver suggested by AIC analysis is added between parenthesis for MAVEN and MEX.

The AIC approach applied to linear regression models ‐ see below for power law models ‐ for MAVEN and MEX thus confirms the main conclusions brought in the previous sections, with EUV fluxes and magnetosonic mach number (for MAVEN) being the major drivers whose removal imply very strong loss of information, with the *θ*_*bn*_ angle of the IMF on a similar level as well, before crustal fields (angular distance to or *ϕ*_*MSO*_ MSO longitude of the strongest crustal source region, or even crustal magnetic field pressure when included in AIC) and solar wind dynamic pressure at a comparable level. Regarding these two, the ranking differs between MAVEN and MEX, with angular distance ranked above solar wind dynamic pressure for MEX but after it in the MAVEN case (thus confirming that lower direct correlations do not necessarily mean reduced influence, in particular due to complex cross‐correlations). *ϕ*_*MSO*_ appears less than or as influent as the angular distance depending on the mission. The IMF intensity and clock angle are also parameters of influence whose removal lead to information loss, but less than other parameters. This is also the case for the equator to pole asymmetry in the MSE frame discussed in Section 3.3, estimated by the absolute value of the cosine of the ”clock angle” shown in Figure 6, that is, the angle of the BS crossing from the IMF vector in the terminator plane.

Moreover, the *θ*_*bv*_ and *θ*_*vn*_ parameters removal do not imply information loss (compared to a simple constant) and are thus considered not significant by AIC analysis.

The use of power law functional forms instead of linear relationships leads only to slight ranking changes for the MAVEN least influent parameters (*θ*_*vn*_ and *θ*_*bv*_). One can note that linear or power law dependence laws are widely considered for EUV, magnetosonic Mach number, or crustal field pressure, but it is difficult to anticipate functional forms for the angular characteristics of the IMF, however, the robustness of the AIC results for both linear and power law models and their coherence with more direct previous analysis suggests the ranking proposed is consistent. Moreover, in first order, most of the relationships can be considered linear (or power laws) which makes the linear model a weak assumption.

#### 3.5.2 The LASSO Regularization

As a complementary approach to the AIC results, we performed an analysis with the Least Absolute Shrinkage Selection Operator method (LASSO; see a detailed description in Appendix B) which is another model selection approach. LASSO is a commonly used supervised regularization method (Tibshirani, 1996), where a penalty term allows the identification of the significance of predictors in a regression model, with possible cross correlations. Compared to classical regression, the coefficients of each variable are constrained by the sum of the modulus or absolute values of the coefficients being as small as possible, the sum of the coefficients being multiplied by a regularization parameter *λ*. When increasing the regularization parameter *λ* from zero (i.e., LASSO switched off or the classical multivariate regression), the weight of the sum of the coefficients in the optimization process becomes eventually strong, which leads the algorithm to eventually set to zeros some of the coefficients, thus selecting the most significant variables only. For large *λ* values, more coefficients of the regression are set to zero, thus removing the associated predictors from the model selected.

We used the glmnet R package to compute the LASSO procedure. The variables introduced in the linear regression model are the same as for the AIC procedure, and were at first standardized ($x_{i_{\mathrm{std}}}=\left(x_{i}-m_{i}\right)/\sigma_{i}$ with *m*_*i*_ and *σ*_*i*_ mean and standard deviations of the variables *x*_*i*_) to remove the specific dynamics of each predictor (e.g., cosines range from −1 to 1 while the MEX EUV ranges from 0 to 100) and thus allow to make the regression coefficients comparable to each other with the same mean (0) and standard deviation (1) values for each driver distribution. The definition of the training set to learn from and of the test set and the determination of the best regularization *λ* value is obtained from a cross‐validation procedure with 10‐folds to avoid overfitting.

**Figure 8:**
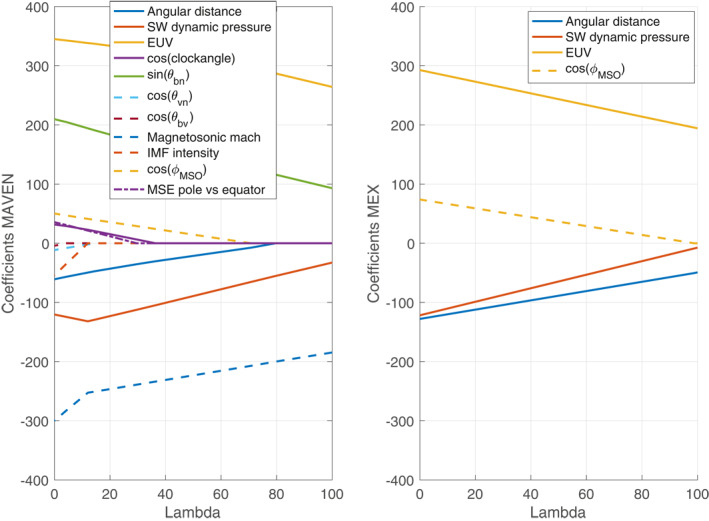
Coefficients of the Lasso regression of the MAVEN (left) and MEX (right) drivers of the Martian shock extrapolated terminator altitude, as a function of the regularization parameter Lambda.

Figure 8 provides the results of the LASSO procedure for both MAVEN and MEX Martian BS crossings datasets. The figure shows the coefficients associated with the variables included in the regression to predict the extrapolated terminator altitude of the BS *R*_*TD*_, as a function of the penalty term *λ*. Low *λ* values in the figure correspond close to the classic regression procedure, while large *λ* values correspond to a strong regularization in the algorithm to select the most important predictors and put the coefficient of the least important to 0. Positive and negative coefficients correspond to the sign of the slope of the *R*_*TD*_ versus the predictors, for example, the BS location increasing for large EUV fluxes (positive coefficient) but decreasing for larger Mms (negative coefficient).

The MEX results show that EUV displays for all *λ* values the largest coefficient among the four variables included (i.e., EUV, SW dynamic pressure, angular distance, and the cosine of *ϕ*_*MSO*_). The SW dynamic pressure and angular distance coefficients are close with no penalty (slightly larger for angular distance), while the SW dynamic pressure coefficient drops more rapidly than the one for angular distance with increasing penalties, reaching zero at large penalties. The coefficient for *ϕ*_*MSO*_ the longitude of the strongest crustal source region is the fourth coefficient in absolute value and reaches 0 at a large penalty.

The MAVEN curves, which include the IMF intensity and orientation parameters, show first that EUV and magnetosonic Mach number are always the largest coefficients. Then the largest coefficients are those of the sine of *θ*_*bn*_, and then of solar wind dynamic pressure, which does not reach zero values at high penalty. The angular distance coefficient is also significant at a low penalty, before the cosine of *ϕ*_*MSO*_. The lowest coefficients are then those of the clock angle and MSE pole versus equator parameter. The lowest coefficients, that are non‐zero with no penalty but are set to zero rapidly by the penalty term are the cosines of the IMF orientation angles *θ*_*bv*_ (cone angle) and *θ*_*vn*_, and to a lesser extent the IMF intensity.

Table 2 provides the final coefficients corresponding to the best model for MAVEN and MEX *R*_*TD*_ predictors. The best model corresponds to small penalty terms (respectively 1.2 and 2.4 for MEX and MAVEN), confirming the significance of most of the variables included in the model according to LASSO. However, if the cross validation procedure leads to non zero coefficients for the least significant variables (*θ*_*bv*_, *θ*_*vn*,_ and IMF intensity), several of the folds lead to zero values of these coefficients (mostly for *θ*_*bv*_, *θ*_*vn*_), making their significance weak or inexistent according to LASSO. One can note that using an initial power law functional form (linearized with a logarithm) for the LASSO regression instead of an initial linear form leads to similar results: this leads to different coefficients but the relative values are very close to the linear form conclusions, as well as the evolution of the coefficients as a function of the penalty term.

**Table 2:** Lasso Regularization Regression Results for the MAVEN and MEX Shock Terminator Altitude

Variable	MAVEN Lasso coefficient	MEX Lasso coefficient
Constant	5155.5	5209.3
cos(*θ*_*bv*_)	−2.7 (11)	
cos(*θ*_*vn*_)	−10.3 (10)	
IMF intensity	−47.3 (7)	
cos(clock angle)	30.7 (9)	
MSE pole versus equator	33.9 (8)	
cos(*ϕ*_*MSO*_)	49.0 (6)	73.0 (4)
SW dynamic pressure	−122.0 (4)	−120.3 (3)
Angular distance	−59.4 (5)	−126.9 (2)
Magnetosonic mach	−293.7 (2)	
sin(*θ*_*bn*_)	207.9 (3)	
EUV	344.2 (1)	291.4 (1)

*Note.* The same ordering as in Table 1 was used. The ranking number of each driver suggested by LASSO analysis is added between parenthesis for MAVEN and MEX.

Overall, the LASSO results are consistent with the previous results based on partial correlations and the AIC approach. The EUV and magnetosonic Mach are the major drivers of the BS location, before parallel versus perpendicular BS (*θ*_*bn*_ angle), and then solar wind dynamic pressure or crustal fields (depending on the dataset). The IMF intensity and orientation angles (mostly the clock angle and MSE pole vs. equator) are predictors of nonnull importance (except probably *θ*_*bv*_ and *θ*_*vn*_) but are weak.

We shall remind that the use of the LASSO approach does not aim at providing precise functional forms of the Martian BS location variability with respect to its drivers, which needs a theoretical understanding of the influence of each predictor but to compare the relative importance of the various possible drivers by assuming at first order a linear regression model (or a power law model).

## 4 Discussion

Based on the previous analyses ‐ direct analysis of the drivers, as well as partial correlations or model selection methods such as AIC or LASSO methods ‐, the statistical study of the Martian BS crossings datasets by MAVEN and MEX shows that the terminator distance of the BS increases:

1. when the magnetosonic Mach number decreases (first order importance): the BS is a fast magnetosonic standing wave induced by the supersonic flow encountering the ionized Mars obstacle, which propagates with a velocity determined by the magnetosonic Mach number; the magnetosonic Mach drives do not only the jump conditions at the shock through Rankine‐Hugoniot relations but also the shape of the BS, in particular, the flaring of the BS with a shock cone inversely proportional to the Mach number according to MHD modeling, which modifies the cross section of the BS and its terminator distance
2. for enhanced extreme ultraviolet fluxes (first order importance): the EUV fluxes increase the ionization rate of both the Martian collisional atmosphere and its exosphere, which adds mass to the solar wind flow through the pickup of ions and thus slows down the solar wind, then creating a larger apparent obstacle that pushes the BS further; the EUV fluxes also heat the atmosphere, thus increasing the ionospheric scale height and expanding the exosphere; this influence of EUV happens at various timescales, from intense brutal solar flares to seasonal variations induced by the eccentricity of Mars' orbit and depending on the hemisphere
3. in the case of perpendicular BS: our statistical analysis of the MAVEN data shows the *θ*_*bn*_ angle between the IMF and the normal to the BS is a significant driver of the Martian BS, with several possible reasons but none being conclusive; the most probable physical process is the expected dependence of the anisotropic wave velocity of fast mode magnetosonic waves on the *θ*_*bn*_ angle that is often mentioned in the Earth's and Venusian BS literature based on MHD modeling (but not on data analysis); however, the anisotropy of the wave speed is expected to be significant mostly for low Mach numbers and in the tail, that is, when and where the BS cross section is maximum, as the travel time of the wave; moreover, our data analysis suggests that the observed influence of *θ*_*bn*_ on the BS is not associated with a significant anisotropy of the wave speed
4. with crustal magnetic fields (second order importance) through two ways: though an influence at the spacecraft location (through the local crustal field pressure or the angular distance from the strongest crustal source region, with a large extent), and through an amplification of this influence all the more than the strongest crustal source region center is located close to noon; the crustal fields may impact through several processes on the BS: they first add internal magnetic pressure, increasing the apparent size of the obstacle, then they modify and induce currents that propagate through the induced magnetosphere, interacting with the magnetosheath plasma and the draping of the IMF around the planet, which in the end pushes further the BS; moreover, when the strongest crustal source region is located close to noon (compared to midnight), the travel time for the magnetosonic wave is reduced allowing the BS standing wave to form further upstream the solar wind flow; finally, G22 revealed the existence of a strong coupling between the crustal field influence on the BS and the ionosphere, through a strong correlation with the Total Electronic Content that is a tracer of the ionospheric variability (due to solar irradiance i.e., the major ionization source of the sunlit ionosphere), but also for the thermosphere‐ionosphere coupling (Sánchez‐Cano et al. [2018]); a part of the crustal field influence on the BS is thus probably related to the increased lifetime (and thus density) of plasma trapped on closed field lines in the crustal magnetic field regions where they are protected from the loss mechanims induced by the solar wind interaction, then increasing the local internal plasma pressure and the size of the apparent obstacle to the supersonic solar wind.
5. with reduced solar wind dynamic pressure (second order importance): the solar wind dynamic pressure is widely known as the major driver of Earth's bow shock, and also plays a significant role at Mars; an increased steady incident pressure indeed compresses the whole induced magnetosphere, and thus pushes the BS closer to the planet, while a variable pressure can also induce more complex phenomena such as transients with Hot Flow Anomalies as observed at Earth (Otto & Zhang, 2021)
6. other variables possibly influence the MAVEN BS location, such as the IMF intensity, clock angle, or other IMF orientation angles that could generate asymmetries of the BS surface; large cone angles may for example, in principle be related to increased $\vec{V}X\vec{B}$ electric fields that accelerate pickup ions and increase the mass loading and thus the size of the obstacle to the solar wind; low clock angles can also be associated to a preferential direction of the electric field depending on the IMF direction, thus inducing asymmetric BS locations; moreover, equator versus pole asymmetries in the MSE frame as identified at Venus can also be related to mass loading effects or to an anisotropic wave velocity (Alexander et al., 1986; Jarvinen et al., 2013); however, the correlations between these possible angular drivers and the Martian BS location are either non significant (e.g., larger shock *R*_*TD*_ values along the convection electric field), or significantly reduced after controlling for the first order importance drivers and for the *θ*_*bn*_ influence with using partial correlations.

We showed in the previous sections how to direct simple analysis can lead to an underestimation or an overestimation of the influence of minor drivers when these are cross correlated with major drivers. The use of specific methods such as partial correlations, AIC or LASSO, allows for an efficient disentangling of the various drivers at play. To check the efficiency of these methods, we performed a simple test: we kept for MAVEN the real driver's datasets (thus keeping the existing cross correlations), but artificially modified the real *R*_*TD*_ dataset with the following procedure: we first perform a regression of the *R*_*TD*_ dataset with respect to the various possible drivers and identify the slope value associated with a specific driver we want to test (e.g., the ”clock angle” of the MSE pole vs. equator influence); then we modified the specific influence of this specific driver, by removing (little influence dataset) from the real *R*_*TD*_ dataset, adding 10 times (strong influence dataset), or adding twice with also noise (noisy moderate influence dataset) the slope associated with this specific driver. When applying the partial correlations, AIC and LASSO methods to these three artificial *R*_*TD*_ datasets (little influence, strong influence, noisy moderate influence), one gets results that are coherent with the expectations: all methods show an increased and coherent ranking of the specific driver for an increased influence of this driver (i.e., for the strong influence dataset compared to the noisy moderate and little influence datasets), while the other drivers influence keeps unchanged. This simple test thus confirms these methods are able to identify any significant driver that impacts the RTD dataset, that is, any driver that shows a recurrent pattern, whatever its nature and its reference coordinate system (in the geographical coordinate system for crustal fields, in the MSO system, in the IMF related coordinate system etc.). These methods are more powerful than direct analysis regarding the detection of minor drivers whose influence may be hidden/underestimated/overestimated based on simple direct analysis due to cross correlations with major drivers.

Parametrizing in detail the BS location as a function of its physical drivers is beyond the scope of this paper since this needs a detailed theoretical understanding of the response of the BS to each driver and to a combination of these drivers that may be inter‐related as demonstrated above. However, previous authors tried to infer functional forms of the influence of the main drivers on the plasma boundaries locations, either from empirical relations or theoretical developments, and it may thus be interesting to compare some of these results with our analysis.

One can in particular compare the LASSO regression results (Table 2) that provide coefficients for each driver to functional forms provided by previous studies. In order to compare the LASSO coefficients $\left(b_{LASSO_{i}}\right)$ with direct slopes of classic regressions (*b*_*i*_ in 2.2), one simply needs to divide the LASSO coefficient of a specific variable by the standard deviation *σ*_*i*_ of the variable (or of its logarithm if a power law function is considered) since the variables are standardized in the LASSO approach: $b_{i}=b_{LASSO_{i}}/\sigma_{i}$.

Regarding the SW dynamic pressure influence, Spreiter et al. (1966) used a $P_{SW}^{-1/6}$ SW dynamic pressure dependence for the nose distance of the Earth's magnetopause and BS, later used at different planets including Mars (Verigin et al., 1993). Crider (2004) suggested a SW dynamic pressure influence on the Martian MPB terminator distance with a softer slope than − 1/6: $P_{SW}^{-0.05}$. In comparison, the LASSO regularization gives power law index values of −0.0596/− 0.0268 for MAVEN/MEX datasets when using a power law functional form $\left(RTD=a\prod_{i}X_{i}^{b_{i}}\right)$, which is close to the Crider (2004) value of −0.05 based on MGS data, or to the MEX only values of −0.02/−0.03 by BH16. Recently, Nemec et al. (2020) compared the influence of several drivers on the MAVEN BS crossings (EUV, SW dynamic pressure and crustal field intensity). They suggested a non‐negligible but small influence of crustal fields, but only local crustal fields were considered, and the magnetosonic mach number or IMF orientation parameters were not investigated, however the power law index suggested −0.06 in their study for the SW dynamic pressure is also comparable to the LASSO conclusions.

The Mach number was also introduced early, with for example, Verigin et al. (2003) who used complex analytical equations for the mach number dependence based on gas dynamics approaches. At Mars, Edberg et al. (2010) suggested a linear relation between the terminator distance of the Martian BS with the *Mms* (i.e., *R*_*TD*_ = −0.1 Mms + 3.3 in Martian radii) based on a proxy of the IMF at Mars in the absence of direct measurement by MEX. The MAVEN LASSO coefficients for a linear model (*RTD* = *a* + *∑*_*i*_ *X*_*i*_ ⋅ *b*_*i*_) lead to a slope for *Mms* of −0.06 that is of the same order as the −0.1 slope by Edberg et al. (2010).

The power law index values derived from the LASSO regularization for the angular distance to the strongest crustal source region are of −0.0280/− 0.0474 for MAVEN/MEX, with a steeper slope for MEX, that is even stronger than the slope for the SW dynamic pressure (−0.0268) while it is the contrary for MAVEN as discussed in the previous sections. If we consider a purely local influence of the crustal fields by taking the local crustal field pressure instead of the angular distance (or equivalently crustal field pressures averaged over large angular distance), the power law index associated is reduced by a factor of 4 and thus becomes smaller (or much smaller for MAVEN) than the *P*_*SW*_ power law index. This is consistent with the Nemec et al. (2020) results who only considered local crustal field pressures with a power law index 3 to 4 times smaller than the value for the SW dynamic pressure.

Our results, when compared with a similar approach, are thus consistent with previous Martian studies that mostly focused on individual or few main parameters of influence at a time and for a specific mission. However, it is clear no simple empirical law can account for the whole dynamics of the BS boundary, due to the complex interplay of the physical processes at work that can eventually not be represented by combined linear or power law functions (in particular the IMF orientation angles such as *θ*_*bn*_), and may be strongly cross‐correlated as shown above. Each driver also possesses its own timescales, for example, EUV impacting in general at larger timescales than the solar wind dynamic pressure or magnetosonic Mach number. Besides, our limited time resolution of the characteristics of the highly dynamic SW could lead to a slight underestimation of their influence on the BS location.

Better understanding of the BS dynamics and in particular how internal stimuli propagate through the Martian induced magnetosphere until the BS would need a full 3‐dimensional modeling approach (as suggested by the results of Gruesbeck et al. (2018)), with dedicated MHD or hybrid modeling including the complex crustal field topology and temporal dynamics. Recently Romanelli et al. (2018) investigated the response of the Martian BS and of the MPB to variable conditions of the solar wind with the global three‐dimensional multispecies parallelized hybrid code LatHyS (Modolo et al., 2016). They considered three stationary simulations with various conditions of solar wind density, magnetosonic Mach number, and velocity, while controlling for constant EUV and IMF orientation conditions, and analyzed the consecutive BS location variability to compare with the extreme conditions encountered during the September 2019 events (see Lee et al. [2018]). Li et al. (2020) also developed a three‐dimensional four species multi‐fluid magnetohydrodynamic (MHD) model to simulate the solar wind global interaction with Mars, where they added or removed an ideal dipole‐like local crustal field model to study the influence of crustal fields on the interaction and on the plasma boundaries. Wang, Xie, et al. (2020) also used 3D MHD modeling simulations to investigate the dynamics of the Martian BS, and proposed a parametric model including seven parameters, suggesting that the *Mms* and SW dynamic pressure were the main drivers of the BS location, but they included neither EUV nor crustal fields.

It would also be interesting to further compare with a consistent method the dynamics of the BS with the dynamics of the inner plasma boundaries (Magnetic Pile‐Up Boundary or Induced Magnetosphere Boundary, Ion Composition Boundary, PhotoElectron Boundary…) since the dynamics of a boundary may impact another boundary, such as at Earth between the magnetopause and the BS (Wang et al., 2016) or at Mars between the Induced Magnetosphere Boundary and the BS (Ramstad et al., 2017).

In parallel to modeling approaches, we plan to use the future machine learning and artificial intelligence techniques to provide automatic catalogs of BS crossings for the MAVEN and MEX missions and thus increase our dataset to reduce the influence of cross‐correlations, and eventually identify complex nonlinear relationships between the BS location (or other plasma boundaries) and external/internal drivers. These techniques are indeed mature and proved efficient in space physics to detect plasma phenomena (see e.g., Karimabadi et al. ;2009]; Nguyen et al. [2019]) or to identify parameters of influence (see e.g., Al‐Ghraibah et al. [2015] or Benvenuto et al. [2018]).

## 5 Conclusions

The recent studies of the Martian environment, thanks in particular to the Mars Express and Mars Atmosphere Volatile EvolutioN missions, underline the complexity of the Martian interaction with the incident solar wind, which shapes the plasma boundaries. The bow shock of the planet is known to depend on extreme ultraviolet fluxes and magnetosonic Mach number, while the influence of other possible drivers is less constrained or unknown such as crustal magnetic fields or the Interplanetary Magnetic Field intensity and orientation.

In this study, we analyzed the influence of a number of possible internal and external drivers of the shock location, based the first time on two missions (MAVEN and MEX). We used consistent methods that allow us to compare both mission's datasets, as well as to take into account the cross correlations between the drivers that can modify the interpretation of the data.

We first analyzed the direct influence of the possible drivers of the shock based on linear correlation coefficients or absolute variations of the shock location. Then, we showed that many of the possible drivers are correlated to each other due to their nature and/or the trajectory of spacecraft. We used a partial correlation approach to investigate in detail the impact of these cross‐correlations on the interpretation of the MAVEN and MEX shock crossings datasets. This approach appears crucial to investigate the influence of second order drivers on the shock that may be linked to each other or to stronger drivers. Finally, we used two known different model selection methods, called Akaike Information Criterion (AIC, see Appendix A) and Least Absolute Shrinkage Selection Operator (LASSO, see Appendix B) to compare the relative importance of the shock drivers. These methods ‐ partial correlations, AIC, LASSO ‐ are able to identify any significant driver that impacts the extrapolated terminator distance of the shock, that is, any driver that shows a recurrent pattern, whatever its nature and its reference coordinate system (in the geographical coordinate system for crustal fields, in the MSO system, in the IMF related coordinate system etc.). These methods are more powerful than direct analysis regarding the detection of minor drivers whose influence may be hidden/underestimated/overestimated based on simple direct analysis due to cross correlations with major drivers.

Based on our analysis, we showed that the major drivers of the Martian shock location are extreme ultraviolet fluxes and solar wind magnetosonic Mach number, while crustal fields (through various parameters) and solar wind dynamic pressure are significant but play a secondary role of similar importance. The results for the magnetosonic Mach number and for the IMF‐related parameters are based only on the MAVEN dataset, due to the absence of a magnetic field for MEX. The analysis of the MAVEN data also shows perpendicular shocks are located at significantly higher distances than parallel shocks, but no conclusive explanation was reached despite a number of possible reasons studied, including an anisotropy fast magnetosonic wave velocity depending on the *θ*_*bn*_ angle that is mentioned in the Earth's and Venusian bow shock literature (Chai et al.,2015; Peredo et al., 1995; Russell et al., 1988). Other variables possibly influence the shock location, such as the IMF intensity, clock angle, or other IMF orientation angles (cone angle *θ*_*vb*_, *θ*_*vn*_) that could generate asymmetries of the shock surface, for example, a possible close to equator versus pole asymmetry in the Mars Sun Electric field frame as identified at Venus and probably related to mass loading effects (Alexander et al., 1986; Jarvinen et al., 2013). However, the correlations between these last possible drivers and the Martian shock location are significantly reduced after controlling for cross correlations with the main drivers of the shock including the *θ*_*bn*_ angle influence. Moreover, no further shocks were observed along the direction of the convective electric field, contrary to suggestions by previous authors based on smaller datasets or proxy methods.

Providing an analytical proxy of the shock location that accounts precisely for the combined influence of its drivers appears unrealistic given the complex interplay of the internal and external physical processes at work, each of them possessing its own timescales. Better understanding of the plasma boundaries dynamics and in particular how internal stimuli propagate through the Martian induced magnetosphere until the bow shock will need a modeling approach including the complex crustal field topology and temporal dynamics, as well as a comparison of the dynamics of the various boundaries.

In future work, we plan to investigate the use of machine learning and artificial intelligence techniques that are efficient to provide automatic catalogs of events (e.g., plasma boundaries) and eventually identify complex nonlinear relationships between the boundary's location and external/internal drivers. These techniques are indeed mature and proved efficient in space physics to detect plasma phenomena or to identify parameters of influence.

## Data Availability

The MGS, MEX and MAVEN datasets of shock crossings are available in the https://doi.org/10.5281/zenodo.6240624 repository. All MGS, MEX and MAVEN instruments calibrated data are available on the AMDA web interface (http://amda.cdpp.eu, in the directory/Parameters/Resources/AMDA Database) as well as on the NASA Planetary Data System (https://pds-atmospheres.nmsu.edu/data_and_services/atmospheres_data/MAVEN/maven_main.html and https://pds-geosciences.wustl.edu/missions/mgs/index.htm) for MAVEN and MGS, and in the Planetary Science Archive (https://www.cosmos.esa.int/web/psa/mars-express) for MEX, with in particular the MARSIS data available at https://archives.esac.esa.int/psa/ftp/MARS-EXPRESS/MARSIS/.

## Appendix A Akaike Information Criterion

The Akaike information criterion (AIC) is a criterion based on information theory widely used to perform model selection (Burnham & Anderson, 2002), by estimating the relative quality of several models for a given dataset. When a model is considered to represent a process generating a dataset, some information is lost by using the model. AIC estimates the relative amount of information lost among several models. This method can be used to compare linear or non‐linear models, as long as the models are being fit to the same data. Moreover, AIC takes into account both the goodness of fit of the model and the complexity of the model, with a penalty that increases with the number of estimated parameters to prevent from overfitting.

We assume we have a statistical model of observed data, where k is the number of estimated parameters in the model and $\hat{L}$ is the maximum value of the likelihood function for the model. Then the AIC value of the model is (Akaike, 1974):

(A1)
$$AIC\;=\;2k-2\ln\left(\hat{L}\right)$$

Among several candidate models fitting the data, the preferred model is the one with the minimum AIC value. Only the relative AIC difference among several candidate models is significant ‐ with differences larger than 2 being significant, and larger than 4 very significant ‐, and there is no significance in the absolute value of AIC. The relative likelihood of a model *i* compared to the best model (with minimum AIC) is given by $\exp\left(AIC_{\min}-AIC_{model_{i}}\right)$.

## Appendix B LASSO

The Least Absolute Shrinkage Selection Operator (LASSO) is a commonly used supervised regularization method for regression developed by Tibshirani (1996). LASSO is an efficient technique for shrinkage and selection method, where a penalty term allows the identification of the significance of predictors in a regression model. Compared to classical regression, the coefficients of the regression are constrained by the sum of the modulus or absolute values of the coefficients being as small as possible. These coefficients can even be set to zero, allowing for selecting the most significant variables depending on the regularization parameter. LASSO is in particular efficient with a large number of predictors included a priori in the model.

We assume our parameter of interest *y* depends at first order on a linear combination of variables *x*_*i*_ with an error term:

(B1)
$$y=\sum_{i}\beta_{i}x_{i}+e$$

LASSO gives as a result the regression coefficients *β*_*i*_ as a function of the regularization parameter *λ*, by minimizing the following term with the glmnet R package:

(B2)
$$J\left(\beta_{i}\right)=1/N\sum_{j=1}^{N}{\left(y_{j}-\sum_{i}\beta_{i}x_{i,j}\right)}^{2}+\lambda \sum_{i}|\beta_{i}|$$

When increasing *λ* from zero (i.e., for LASSO switched off or for the classical solution) to larger values, the regularization term sets more coefficients to zero, thus removing them from the selected model. The LASSO technique needs the definition of a training set to learn from and a test set in order to tune the parameter *λ*. A good *λ* value can be obtained by a cross‐validation procedure, for example, 10‐folds in our study to avoid overfitting.
